# How orange carotenoid protein controls the excited state dynamics of canthaxanthin†

**DOI:** 10.1039/d3sc02662k

**Published:** 2023-09-22

**Authors:** Amanda Arcidiacono, Davide Accomasso, Lorenzo Cupellini, Benedetta Mennucci

**Affiliations:** a Dipartimento di Chimica e Chimica Industriale, Università di Pisa Via G. Moruzzi 13 56124 Pisa Italy benedetta.mennucci@unipi.it

## Abstract

Orange Carotenoid Protein (OCP) is a ketocarotenoid-binding protein essential for photoprotection in cyanobacteria. The main steps of the photoactivated conversion which converts OCP from its resting state to the active one have been extensively investigated. However, the initial photochemical event in the ketocarotenoid which triggers the large structural changes finally leading to the active state is still not understood. Here we employ QM/MM surface hopping nonadiabatic dynamics to investigate the excited-state decay of canthaxanthin in OCP, both in the ultrafast S_2_ to S_1_ internal conversion and the slower decay leading back to the ground state. For the former step we show the involvement of an additional excited state, which in the literature has been often named the S_*X*_ state, and we characterize its nature. For the latter step, we reveal an excited state decay characterized by multiple timescales, which are related to the ground-state conformational heterogeneity of the ketocarotenoid. We assigned the slowly decaying population to the so-called S* state. Finally, we identify a minor decay pathway involving double-bond photoisomerization, which could be the initial trigger to photoactivation of OCP.

## Abstract 2

We use nonadiabatic simulations to investigate the early excited-state dynamics of canthaxanthin in the Orange Carotenoid Protein (OCP), providing insight into the observed spectroscopic features and finding a possible photoactivation path.

## Graphical abstract

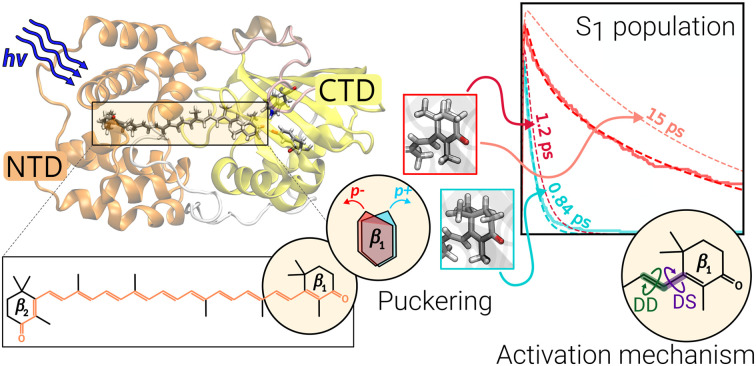

## 1. Introduction

The Orange Carotenoid Protein (OCP)^1,2^ is a water-soluble protein found in photosynthetic cyanobacteria where it plays a fundamental role in their photoprotection mechanism.^3,4^ OCP is able to sense the high-light conditions through its chromophore, a keto-carotenoid embedded between the two protein domains, one C-terminal (CTD) and one N-terminal (NTD). The initial excitation of the carotenoid is followed by a translocation of the chromophore in the NTD. This motion initiates a series of conformational changes in the protein's structure, that goes from a “dark adapted” resting form (OCP^O^) to a “light adapted” active one (OCP^R^).^5–9^ It is the latter OCP^R^ form that can quench the excess excitation energy absorbed by the light-harvesting apparatus of cyanobacteria, namely the phycobilisome (PBS), when the system is exposed to intense light.^10,11^ OCP is able to bind various carotenoids but the crucial feature that determines whether the complex is responsive to light is the possibility of the carotenoid to form hydrogen bonds with two residues of the CTD, Tyrosine 201 (Y201) and Tryptophan 288 (W288). Therefore, the presence of a carbonyl group in the carotenoid is necessary for the protein to be photo-active, even though it is not essential for a stable binding.^12^ Native OCP can bind either echinenone (ECN), 3′-hydroxy-echinenone (hECN) or canthaxanthin (CAN).^13^

In the last few years, various experimental and theoretical studies have clarified many aspects of the translocation of the carotenoid from the initial binding pocket shared between the CT and NT domains and the final domain separation.^7,14–18^ The OCP^O^ → OCP^R^ conversion is a multi-step process,^19^ starting with the rupture of the hydrogen bonds in the CTD.^9,20,21^ This event results in the first stable product of OCP photocycle in a ps time scale, even though its atomistic mechanism is still under debate.^7,15,20^ Indeed, what is still largely unknown is the photochemistry that follows the initial π → π* excitation of the carotenoid and how its time evolution can trigger such radical conformational changes in the protein.

What it is expected for a (keto)carotenoid is that the initial photo-absorption induces a transition to the lowest energy bright state (1B_u_^+^, using a simplified *C*_2h_ symmetry), which rapidly decays to a lower energy dark state (S_1_ or 2A_g_^−^ pseudo-symmetry^22^). This is known to be an ultrafast process that occurs within few hundred fs. For several carotenoids, including CAN and hECN, spectroscopic signatures from another state were observed in the ultrafast process, and this state was called S_*X*_.^23–27^ The final decay of S_1_ to S_0_, which is commonly completed on a time scale of several ps, is even more complex and still openly discussed. It has been suggested that other dark states are involved. One is generally referred to as S*,^16,27–32^ while the other is an intramolecular charge transfer (ICT) state, strongly coupled with S_1_.^5,14,15,33–36^ These states have different decay times. For keto-carotenoids a faster decay to the ground state occurs on the scale of few picoseconds, generally associated with the 2A/ICT decay, while a slower component of tens of picoseconds is attributed to S*. The nature of the couplings between those states is still unknown, along with the nature of S*.

In this context, an explicit simulation of the excited state dynamics of the ketocarotenoid in OCP could represent a powerful approach to disentangle the different decay pathways towards the ground state. However, an accurate *ab initio* QM description of the electronic structure of (keto)carotenoids is very challenging. The multireference character of their electronic states, in fact, calls for very expensive computational methods, whose applicability is still limited to small molecules. To overcome these limits, an effective strategy is to adopt a semiempirical QM method in combination with a multiconfigurational description of the electronic states, as well as to introduce the effects of the protein and the solvent through a molecular mechanics (MM) model; this integrated approach has been successfully used in previous studies of carotenoids.^18,37^ In the present work, we employ this strategy to reproduce the multi-step decay pathway of canthaxanthin in OCP. This is obtained by applying QM/MM nonadiabatic excited-state dynamics based on the surface hopping (SH) method.^38^

Such a computational approach allowed us to simulate both the ultrafast S_2_ → S_1_ conversion and the slower S_1_ decay towards the ground state. In the ultrafast relaxation process, we observed the formation of an intermediate “dark” state of B_u_^−^ pseudo-symmetry, which we associated with the spectroscopic S_*X*_ state, similar to what we found for lutein in a previous study.^37^ Regarding the S_1_ → S_0_ conversion, our simulations indicated that the decay of the S_1_ population is multi-exponential and the different excited-state lifetimes stem from the ground-state structural heterogeneity of CAN in OCP. In particular, we assigned the slow component of this decay to the S* state. Finally, we found a minor S_1_ → S_0_ decay pathway involving the isomerization of a double bond of CAN adjacent to the β_1_ ring in the CTD. This photoisomerization could be the trigger of OCP light activation.

## 2. Methods

### 2.1. Electronic states

The electronic states of CAN were computed using the semiempirical configuration interaction method FOMO-CI.^39–41^ In particular, we used the AM1 form of the semiempirical Hamiltonian,^42^ with the parameters optimized for lutein in our previous work.^37^ In the FOMO-CI calculations we employed an active space of 8 electrons in 10 molecular orbitals of π type, a Gaussian energy width for a floating occupation of 0.075 Hartree, and a determinant space including all single and double excitations within the orbital active space. Additional information regarding the selection of the orbital active space for the FOMO-CI method is provided in Section S1.†

In the simulations of CAN in OCP, we used a quantum mechanics/molecular mechanics (QM/MM) scheme,^43^ in which CAN, treated at the semiempirical QM level, is embedded in the MM protein environment of OCP. For the MM part, we employed the parameters of the ff14SB AMBER force field.^44^ The interaction between the QM and MM subsystems was treated using the electrostatic embedding scheme. In all the QM/MM simulations, we considered a subsystem of the solvated OCP^O^ as investigated in ref. 45. Specifically, in the MM part we included only the protein residues, water molecules and ions within a distance of 25 Å from CAN. Furthermore, in the molecular dynamics simulations, only the MM residues within 18 Å of CAN were allowed to move, while all the other MM atoms were frozen.

### 2.2. Nonadiabatic excited-state dynamics

The nonadiabatic excited-state dynamics of CAN was simulated using the “fewest switches” surface hopping (SH) method.^38,46^ The starting conditions (*i.e.*, nuclear coordinates and velocities, and the initial electronic state) for the SH simulations were sampled from ground-state thermal trajectories at 300 K that we performed with the Bussi–Parrinello stochastic thermostat.^47,48^ In particular, for the simulations in OCP we sampled the last 5 ps of ten QM/MM equilibrations propagated for 10 ps, while for the gas phase simulations we used the last 20 ps of a single QM thermal MD. More details on the ground state equilibrations are provided in the ESI (see Section S2 in the ESI†). In the sampling procedure, the initial electronic state was selected according to the radiative transition probability from the ground state,^41^ within an excitation energy range of 2.63 ± 0.15 eV for CAN in OCP, and 2.67 ± 0.15 eV for CAN in the gas phase. In both cases, the selected energy window includes most of the main band of the UV/vis absorption spectrum of CAN, computed along the thermal equilibrations (Fig. S15 in the ESI†).

In the SH simulations, the time evolution of the electronic wavefunction was computed by making use of a locally diabatic representation,^40,49^ and with a time step of 0.2 fs, employed in the integration of both the nuclear degrees of freedom and the electronic ones. Quantum decoherence effects along the SH trajectories were approximately taken into account using the overlap decoherence correction (ODC) scheme, with the following parameters: *σ* = 1.0 a.u. (Gaussian width) and *S*_min_ = 5 × 10^−3^ (minimum overlap).^50,51^ For the simulations in OCP, a total of 364 SH trajectories (39 starting from S_1_, 318 from S_2_, and 7 from S_3_) were simulated, while 39 SH trajectories, starting from the S_2_ state, were computed in the gas phase simulations. All the SH trajectories were propagated for 20 ps, under energy-conserving conditions. The six lowest singlet states were taken into account in the nonadiabatic SH dynamics, which was considered sufficient *a posteriori*, given that states above S_3_ are never populated. For each simulation time, the population of each electronic state *i* was computed as the fraction of SH trajectories running on the *i*-th PES.

All the simulations were performed using a development version of the MOPAC code,^52^ interfaced with the TINKER 6.3 (ref. 53) molecular modeling package, in which the SH method and the QM/MM semiempirical FOMO-CI technique were implemented.

## 3. Results

### 3.1. Ultrafast S_2_ → S_1_ dynamics

We first analyze the ultrafast component of the photoinduced dynamics of CAN, namely the internal conversion between S_2_ and S_1_. This process is known to occur in the subpicosecond time scale, therefore we focus on the first 200 fs after photoexcitation (Fig. 2), and follow the time evolution of the adiabatic state populations for CAN in the gas phase and in OCP. In both cases, after vertical excitation, we observe rapid oscillations of the populations of S_2_ and S_3_ within the first 100 fs, indicating a back-and-forth population exchange between these two states. This is a symptom of multiple recrossings between two potential energy surfaces, favored by the near degeneracy of S_2_ and S_3_ (the energy profile is reported in Fig. 2, panels B and B′, while the energy gap distribution at S_2_ → S_3_ and S_3_ → S_2_ transitions is shown in ESI Fig. S17†). Analogous behaviour was observed for the ultrafast dynamics of Lutein,^37^ and was ascribed to the crossing between states of 1B_u_^+^ and 1B_u_^−^ character. These states correspond to S_2_ and S_3_ respectively at the Franck–Condon point, but they continuously swap along the dynamics. The S_2_–S_3_ population recrossing is less pronounced in OCP, compared to the gas phase, likely due to the larger S_2_–S_3_ energy gap after the first 10 fs of dynamics. The nuclear dynamics within the first 100 fs is dominated by strong oscillations of the bond-length-alternation (BLA) coordinate (Fig. 2, panels C and C′), which modulates the excitation energy of states S_1_–S_3_. This allows S_2_ and S_3_ to become very close in energy at regular intervals. Finally, the BLA oscillations are damped after ∼100 fs, although the damping time seems shorter in OCP.

To follow the population dynamics in the ultrafast timescale, we analyzed the potential energy curves of the first four excited states of CAN along the BLA coordinate (Fig. S11 in the ESI†). Due to the qualitatively similar picture obtained *in vacuo* and in OCP, this analysis has been performed for the isolated molecule. Vertical excitation populates 1B_u_^+^ (S_2_), and then CAN rearranges its geometry towards smaller BLA values, following the excited-state gradient. Since the energy difference between 1B_u_^+^ and 1B_u_^−^ is small, the nuclear wavepacket can oscillate back and forth along the BLA coordinate, encountering the crossing region between these two states (see Fig. 2). Although part of the population is transferred to 1B_u_^−^, the majority remains diabatically in the 1B_u_^+^ state, which becomes S_3_ at small BLA values (Fig. S11 in the ESI†). This explains why the adiabatic state S_3_ is periodically populated, and the oscillations of adiabatic populations perfectly match the oscillation period of the BLA.

The 1B_u_^−^ minimum is close to the S_1_ one in the BLA picture (Fig. S11 in the ESI†), therefore the transition to 1B_u_^−^ might facilitate the decay to S_1_ in an ultrafast time scale. As CAN settles in the S_1_ state, the BLA drops to nearly zero (Fig. 2), indicating that single and double bonds have similar strength in this state. This reflects the electron density displacement from double to single bonds in the excited states of CAN, as this displacement is more substantial for the S_1_ state.^18^ Along the ultrafast dynamics, other degrees of freedom need to be activated in order to reach the 1B_u_^−^ and finally the S_1_ state. These include both all other C–C and C=C modes and out-of-plane torsional motions. The latter are enhanced in OCP, as CAN's geometry is strongly distorted due to the constraints exerted by the protein pocket. This also explains why the initial decay to S_1_ is slightly faster in OCP than in the gas phase.

Comparing the population evolution in the adiabatic and diabatic representations (Fig. S12 in the ESI†), we can see that the S_2_/S_3_ population oscillations persist for the lifetime of the 1B_u_^+^ state. The 1B_u_^+^ bright state is thus depopulated in <100 fs in the gas phase and in <50 fs in OCP, which is consistent with the ∼20 fs emissive lifetime extracted from fluorescence experiments.^54^ In contrast to ref. 54 though, we find that the ultrafast decay of 1B_u_^+^ is mediated by the transfer to 1B_u_^−^, before the S_1_ state is reached.

The results obtained indicate a role of the 1B_u_^−^ dark state in the S_2_ → S_1_ relaxation process, as observed previously for Lutein.^37^ This bridge state was also detected experimentally for different carotenoids and it is often referred to as S_*X*_.^23–27^ The relaxation pathway S_2_ → S_*X*_ → S_1_ is shared between the gas-phase and OCP simulations, with a faster conversion between S_2_ and S_*X*_ observed in OCP. This result, and the striking analogy to the case of Lutein, suggest that the S_*X*_-mediated decay pathway could be a rather common feature in the photoinduced dynamics of carotenoids.

### 3.2. The S_1_ → S_0_ relaxation process

We now consider the slower part of the decay process, up to the first 20 ps. In Fig. 3A and B we again compare the populations of the adiabatic states of CAN in the gas phase and in OCP.

In the gas phase simulations, the population of S_1_ increases within the first picosecond and then decays to S_0_ with a single-exponential behaviour. We extracted the rise and decay times of S_1_ assuming the following kinetic model:1
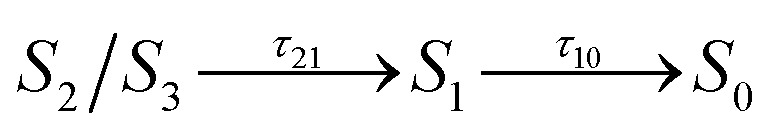
where *τ*_10_ and *τ*_21_ are the lifetimes of S_1_ and S_2_ + S_3_, respectively. Summing the populations of S_2_ and S_3_ allows us to avoid dealing with the population oscillations described above. By directly fitting the population of S_1_ (eqn (8) in the ESI†) we obtained *τ*_21_ = 0.13 ps and *τ*_10_ = 4.8 ps.

In contrast to gas-phase simulations, in OCP the decay of S_1_ cannot be described by a single-exponential function (Fig. 3Band C). To fit the S_1_ population, we used a similar kinetic model (eqn (1)), but with two decay times for S_1_, namely *τ*_A_ and *τ*_B_:2
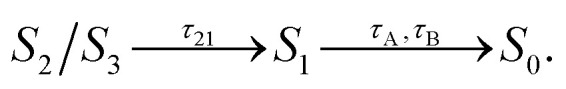


The decay components *τ*_A_ and *τ*_B_ are weighted respectively by *w*_A_ and *w*_B_ = 1 − *w*_A_ (eqn (9) in the ESI†). The fit yielded *τ*_21_ = 0.1 ps, and the following S_1_ lifetimes: *τ*_A_ = 14.3 ps and *τ*_B_ = 0.8 ps, with weights 40% and 60%, respectively. A weighted average gives us a lifetime of 6.2 ps. Although both decay times of S_1_ in OCP differ significantly from the one obtained in the gas-phase simulations (4.8 ps), the average lifetime is only slightly longer. However, the two substantially different lifetimes obtained in our simulations strongly suggest a heterogeneity within OCP that is not present in gas-phase simulations.

**Fig. 1:**
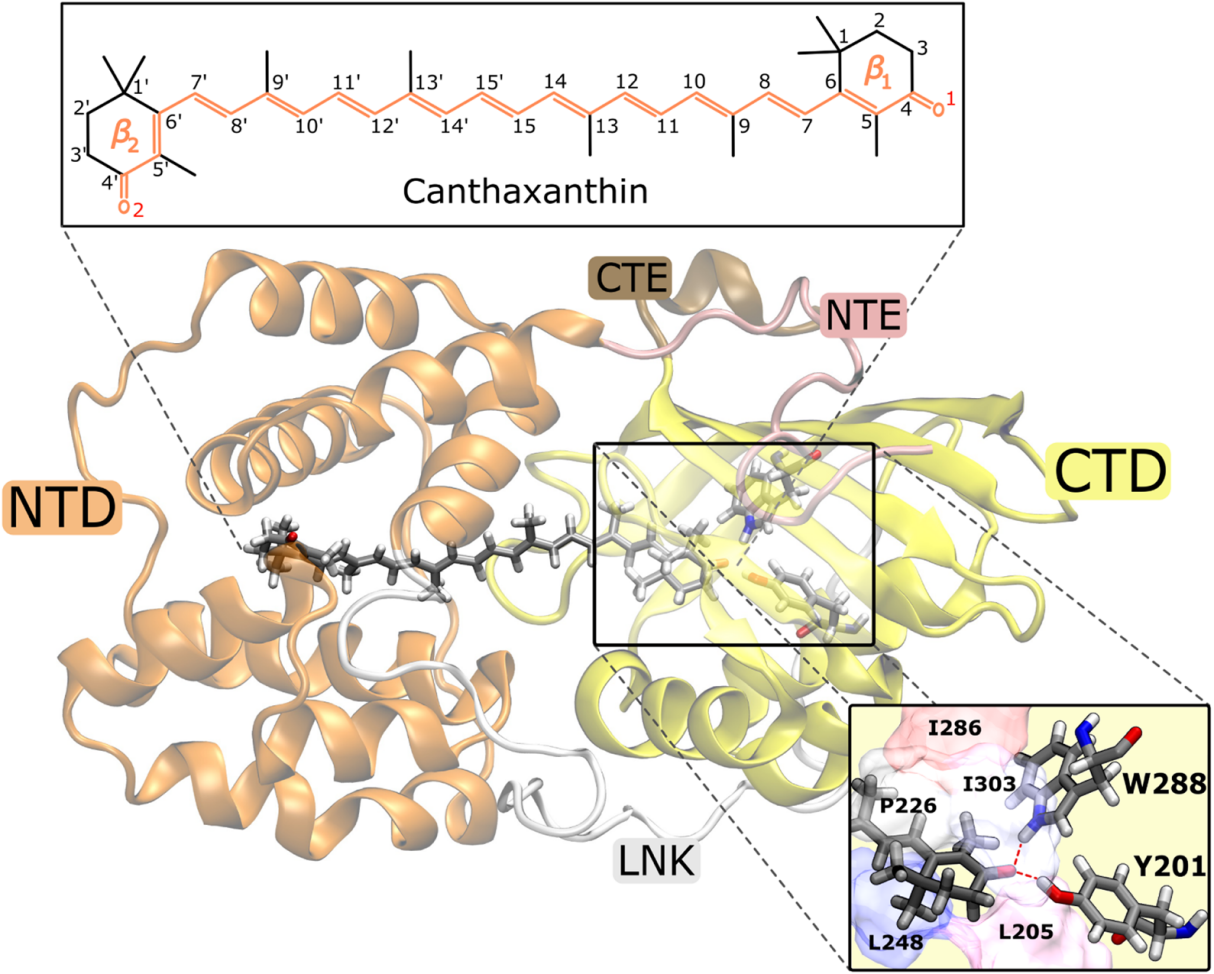
Representation of the OCP^O^ structure. The keto-carotenoid canthaxanthin (CAN) is embedded between the C-terminal (CTD) and the N-terminal (NTD) protein domains. A view of the hydrogen bonds between CAN and the two CTD protein residues Tyrosine 201 (Y201) and Tryptophan 288 (W288) is also shown.

To investigate the origin of such different S_1_ lifetimes in OCP, we analyzed the structural features of CAN in the ground-state ensemble, namely at the starting geometries for the SH trajectories (*t* = 0). From this analysis, we noticed that the initial geometries can be divided into two groups according to the puckering of the β_1_ ring of CAN, located in the CTD (Fig. 1) (see details in Section S6 in the ESI†). Notably, ring puckering was recognized to be a determinant of heterogeneity in OCP.^45,55^ Here we assign the structures with *ϕ* ≤ 0° to the p^−^ conformation (52% of the total number of trajectories), and all the other structures (with *ϕ* > 0° at *t* = *t*_0_) to the p^+^ conformation (Fig. S14 in the ESI†). We then divided *a priori* the SH trajectories on the basis of the puckering angle at the initial time.

**Fig. 2:**
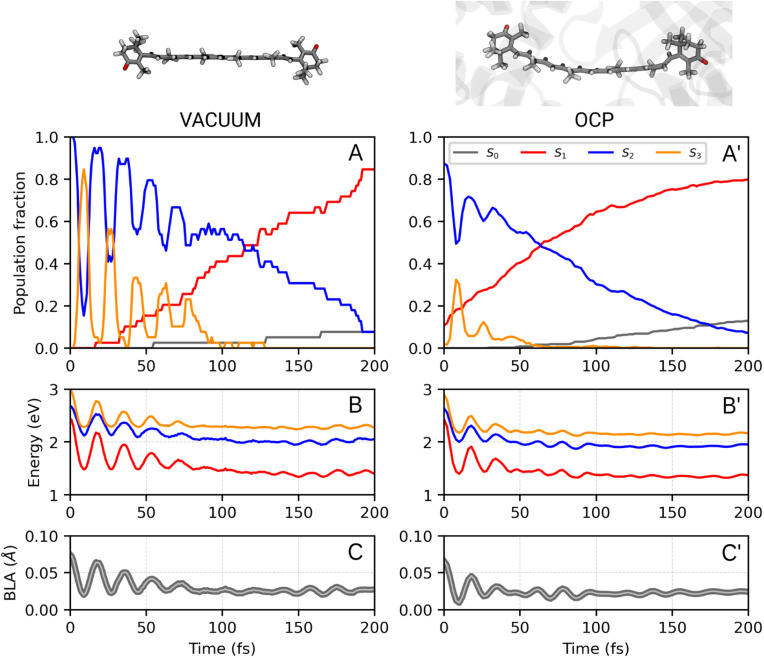
Ultrafast portion of CAN excited-state dynamics *in vacuo* (A, B, C) and in OCP (A′, B′, C′) (see ESI Fig. S12† for the diabatic representation of the vacuo populations and energies). Results are obtained from 39 trajectories and 364 trajectories *in vacuo* and in OCP, respectively. Panels A/A′ show the adiabatic state populations of CAN during the first 200 fs of the SH simulations. In panels B/B′ and C/C′, respectively, the excitation energies from the ground state and the BLA values are reported.

**Fig. 3:**
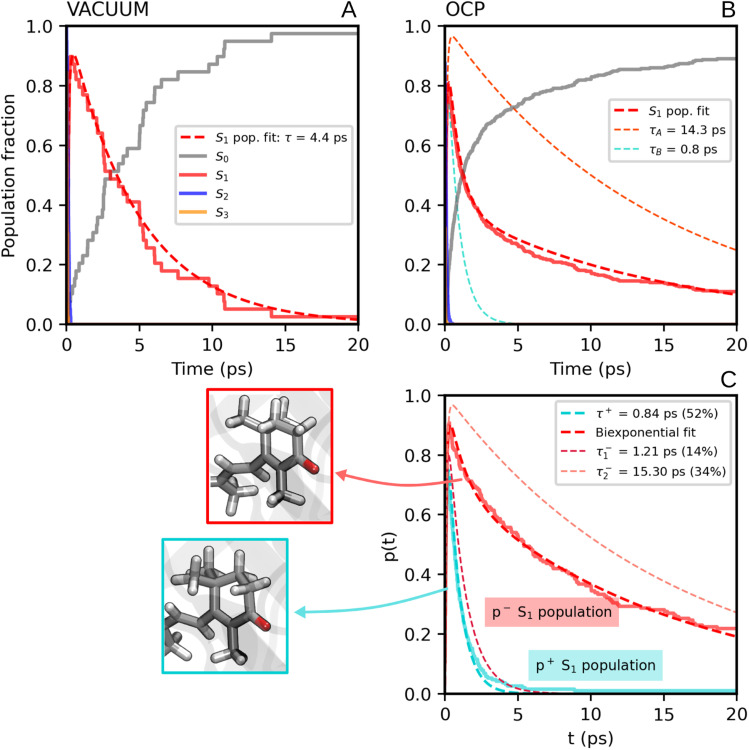
(A) Populations of the adiabatic states of CAN obtained from the SH simulations in the gas phase. The red dashed line is the fitting function for the S_1_ population (eqn (1) and S8 in the ESI†). (B) Populations of the adiabatic states of CAN for the SH simulations in OCP, with the fitting biexponential function for the S_1_ population (eqn (2) and S9 in the ESI†) reported as a dashed red line, together with its single-exponential components (light-blue and orange dashed lines). (C) Population of the S_1_ state for the two groups of SH trajectories obtained from the puckering-based separation (see text). The single-exponential fitting function for the p^+^ group of trajectories and the bi-exponential fitting function for the p^−^ group, in addition to its single-exponential components, are also reported (dashed lines).

In Fig. 3C, we report the time evolution of the S_1_ population for the two groups of SH trajectories, p^−^ and p^+^. A clear trend appears, as the decay for the p^+^ group is much faster than the p^−^ trajectories. The S_1_ population in p^+^ trajectories can be fitted by a single exponential decay with a time constant of 0.84 ps, close to the fastest time constant of the entire ensemble *τ*_A_. On the other hand, the population for the p^−^ group shows again a bi-exponential decay, from which we extracted two lifetimes, namely 1.21 ps and 15.30 ps (with relative weights of 0.3 and 0.7, respectively). The longest time constant is again close to the slowest decay time *τ*_B_ obtained for the entire ensemble.

Separating the SH trajectories on the basis of the ground-state conformation allows a clear discrimination between the faster decaying trajectories from the slower ones. In turn, this indicates that the multiexponential behaviour of the S_1_ population decay is determined by heterogeneity in the ground-state ensemble. Overall, three decay times for S_1_ can be extracted: a sub-picosecond lifetime of 0.8 ps obtained from the p^+^ group, and two longer decay times of 1.2 ps and 15.3 ps, both obtained from the p^−^ trajectories.

### 3.3. Structural changes of CAN during the excited-state decay

**Fig. 4:**
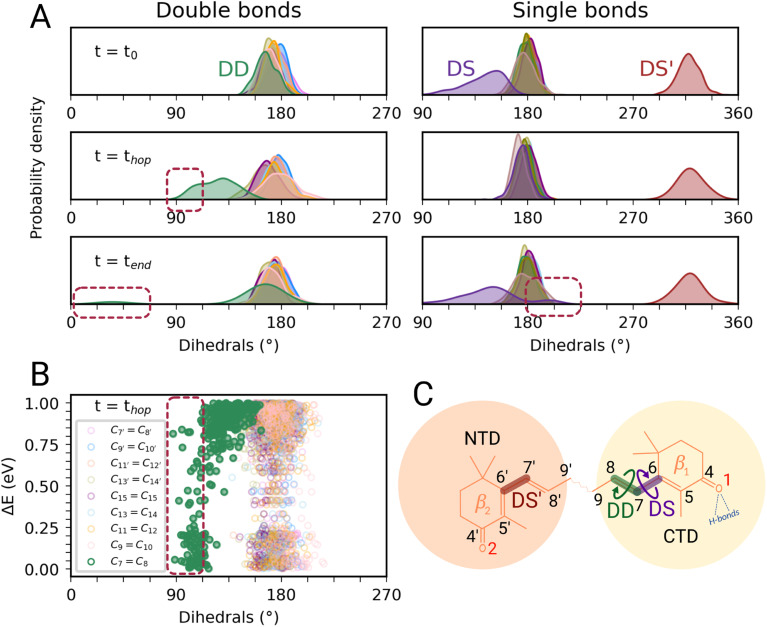
Torsional motion during the excited-state dynamics of CAN in OCP. (A) Complete overview of the dihedral values of CAN's π-conjugated chain along the SH simulations. In the left panels, the distributions of double bond dihedrals are reported, while on the right all the distributions of single bond dihedrals are shown. From up to down, different times of the SH simulations are considered: the initial conditions (*t* = *t*_0_), the S_1_ → S_0_ hops (*t* = *t*_hop_), and the final time reached by each trajectory (*t* = *t*_end_). (B) Energy gap between *S*_1_ and *S*_0_*versus* the C=C dihedrals of CAN at the S_1_ → S_0_ hops. (C) Schematic representation of CAN's far end structure in OCP. The three main dihedrals are also represented (DS in purple, DS′ in red, and DD in green).

In this section, we characterize the main structural changes of CAN occurring during the S_1_ → S_0_ relaxation process. In Fig. 4A, we report the distributions of the dihedral angles around the C=C and C–C bonds of the π-conjugated chain of CAN for different times of the SH simulations in OCP. Each dihedral angle of CAN was computed as the clockwise angle between the two half-planes defining the dihedral, *i.e.*, all dihedrals range between 0° and 360°. Under the initial conditions (Fig. 4A, panel *t* = *t*_0_), we find a significant distortion for the single bond connecting the π-conjugated chain with the β_1_ ring of CAN. This bond is in an s-*trans* conformation but the corresponding dihedral angle (from now DS) ranges from 180° to almost 90°. The opposite dihedral (DS′, Fig. 4C), involving the β_2_ ring in the NTD, displays instead an s-*cis* conformation with values between 300° and 360°. The dihedrals around all the other C–C and C=C bonds of the π-conjugated chain of CAN assume values between 150° and 210°, typical of a planar all-*trans* geometry.

During the nonadiabatic dynamics, the most significant structural changes of CAN can be observed at the S_1_ → S_0_ transitions. These occur at different times depending on the trajectory, but are analyzed together in Fig. 4A (middle panel *t* = *t*_hop_). The distribution of the dihedral around the C_7_=C_8_ double bond (DD from now on, Fig. 4C) is significantly broader and partially shifted towards 90°. On the other hand, the adjacent single bond dihedral (DS) presents a narrower range of values, closer to the dihedrals around the other conjugated C–C bonds (Fig. 4A). This occurs because, in the S_1_ geometry, DD and DS exchange their double-bond character and consequently their flexibility. As a consequence, the significant distortion around the β_1_ ring forces the DD dihedral to twist as soon as the DS dihedral planarizes (Fig. 4C). Conversely, the remaining single and double bonds in the conjugated chain of CAN do not undergo any significant change.

Notably, the distortion of DD is most of the time accompanied by a significant decrease in the adiabatic energy gap between S_1_ and S_0_ (Δ*E*, Fig. 4B). By looking at the distribution of Δ*E* at the hopping times of the SH trajectories (Fig. S16 in the ESI†), we can notice that for many trajectories the S_1_ → S_0_ transition takes place with a very small energy gap (Δ*E* < 0.25 eV). This indicates the reaching of a S_1_/S_0_ crossing region when DD approaches 90° (Fig. 4B), which promotes internal conversion to S_0_. Such a crossing is not observed in gas phase simulations, where the S_1_ → S_0_ hops always occur with Δ*E* > 0.7 eV and without any particular change in CAN's structure. In OCP, the orientation of the β_1_ ring likely facilitates distorted structures when CAN is in the S_1_ state. As a consequence, CAN is able to access structures where the S_1_ and S_0_ states are close in energy.

**Fig. 5:**
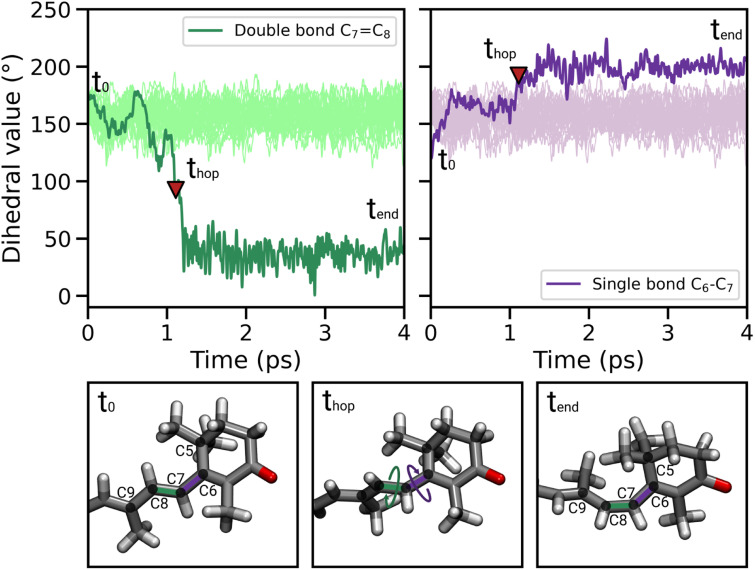
Illustration of the photoisomerization mechanism of DD and DS for a representative reactive trajectory, accompanied by selected snapshots for different simulation times, *i.e.*, before (*t*_0_), during (*t*_hop_) and after (*t*_end_) the isomerization. The red triangle corresponds to the S_1_ → S_0_ hop. The light green and light purple lines represent the DD and DS values along the SH trajectories in which the isomerization does not occur.

In most of the SH trajectories, after the S_1_ → S_0_ transition, DD and DS go back to their original values (Fig. 4A, panel *t* = *t*_end_). However, for a small number (∼12%) of trajectories we observe that DD reaches values close to zero, indicating *trans*-to-*cis* isomerization around the double bond, accompanied by a torsion around DS towards the s-*cis* conformation. This isomerization is illustrated in Fig. 5 for a representative SH trajectory. From the opposite behavior of the two adjacent single and double bonds, we identify the isomerization mechanism as a *hula-twist* mechanism, a volume-conserving photoisomerization already observed in several protein-bound chromophores, such as retinoids, cryptochromes, and phytochromes.^56,57^

**Fig. 6:**
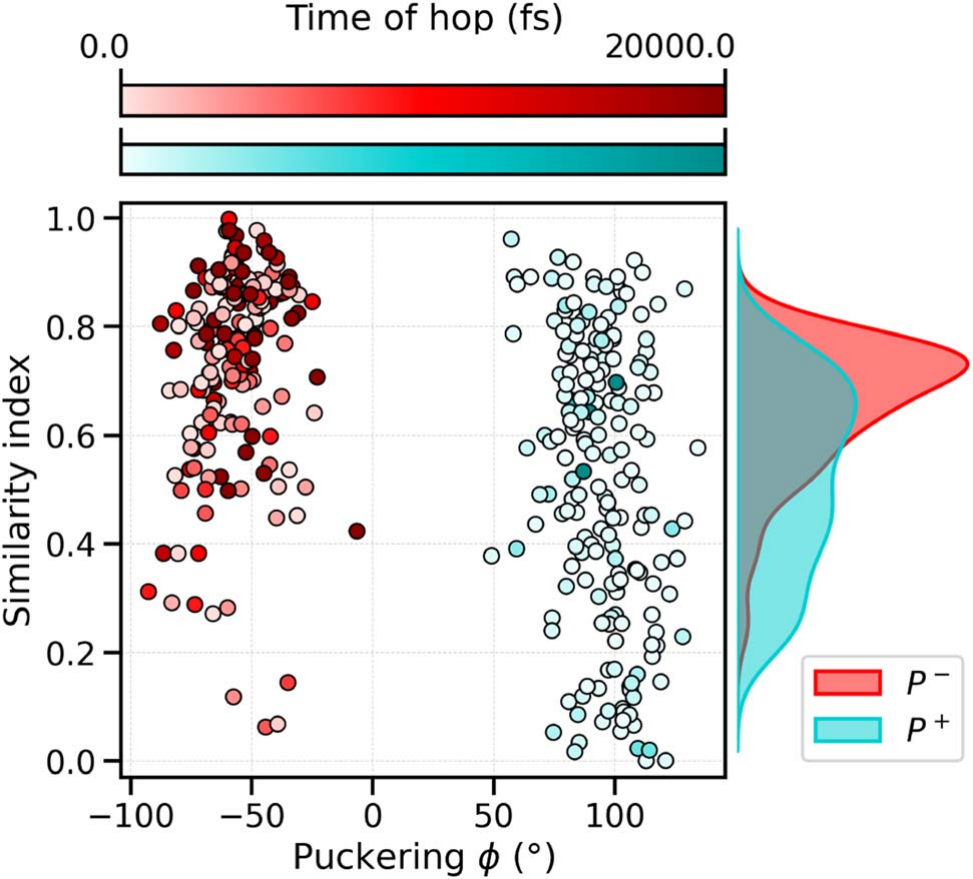
Correlation between the puckering conformation, the distortion of DS under the initial conditions and the hopping times of the SH trajectories. The red points belong to the p^−^ cluster, the light-blue ones to the p^+^ cluster. The color gradient underlines the hopping time in the corresponding SH trajectories, where the darker dots are characterized by a late decay or no decay at all in the 20 ps of simulation time, while the lighter dots are characterized by an early decay.

To analyze what determines the likelihood of photoisomerization, we divide the trajectories on the basis of the puckering angle (see Section 3.2). Most of the photoisomerization events occur in the p^+^ trajectories (Fig. S14 in the ESI†), with a yield of 18%, while the photoisomerization yield for the p^−^ group is only 5%. The higher yield of isomerization and the faster decay of the S_1_ population obtained for the p^+^ trajectories can be both related to the initial (*t* = *t*_0_) distortion of DS, which we evaluated using the similarity index, *s*_θ_ = cos^2^ *θ*. *s*_θ_ ranges between 0 and 1, 0 corresponding to the twisted conformation (*θ* = 90°) and 1 corresponding to the perfectly planar conformation, either s-*cis* (*θ* = 0°) or s-*trans* (*θ* = 180°). As we can see from Fig. 6, the distribution of *s*_DS_ for the initial geometries of the p^+^ trajectories is significantly broader and shifted towards smaller values, compared to the corresponding distribution for the p^−^ group. The mean values at *t* = 0 are *s*_DS_ = 0.51 for p^+^ and *s*_DS_ = 0.73 for p^−^, indicating a larger initial deviation of DS from the perfectly planar s-*trans* conformation. The p^+^ trajectories are also associated with a smaller S_1_–S_0_ energy gap at hops, and a larger twisting of DD at the same S_1_ → S_0_ transition geometries (see Table S2†). Therefore, the larger initial distortion of DS, observed in the p^+^ trajectories, increases the accessibility of highly distorted molecular geometries, where the twisting around DD is large and the S_1_–S_0_ energy gap is small, leading to both a faster S_1_ → S_0_ decay and a higher photoisomerization yield.

To investigate the origin of the larger initial distortion of DS in the p^+^ trajectories, we analyzed the arrangement of the amino acid residues surrounding the β_1_ ring of CAN in the ground-state ensemble. Specifically, we computed the minimum distances between the center of the β_1_ ring of CAN and the following residues of the CTD: L205, L248, I303, P226, and I286 (Fig. 1). The C_2_ atom was excluded from the definition of the center of the β_1_ ring to avoid bias coming from the puckering itself. Moreover, the two hydrogen-bonded residues Y201 and W288 (Fig. 1) were not included because their minimum distance from the β_1_ ring of CAN is not affected significantly by the puckering. From our analysis, it turns out that residues I303, P226, and I286 are generally closer to the β_1_ ring in the p^+^ geometries, while for both L248 and L205 the distance from the β_1_ ring is shorter in the p^−^ geometries (Fig. S18 in the ESI†).

To obtain a more direct visualization of the correlation in the ground-state ensemble between the puckering, the disposition of the aforementioned amino acids, and the value of *s*_DS_, we report in Fig. S19 in the ESI† a principal component analysis (PCA) based on the distances from the 5 selected residues. From the left panel of Fig. S19† it is evident that the puckering value follows the separation given by the PC1, with very few exceptions. In addition, at negative values of the PC1, *s*_DS_ reaches lower values, *i.e.*, geometries that are farther from the planar conformation, showing that the distortion is linked to the disposition of the residues.

Combining all these analyses, we conclude that the larger initial twisting of DS in the p^+^ trajectories can be attributed to the different, puckering-dependent, placement of the β_1_ ring inside a rather rigid protein binding pocket.

## 4. Discussion and comparison with experiments

### 4.1. Ground-state heterogeneity and multiexponential decay

Our simulations of CAN in the gas phase indicated a single-exponential decay of the S_1_ population, with a time constant of 4.8 ps. This time is very close to the reported S_1_ lifetimes determined by transient absorption spectroscopy in solvents of different polarities, namely 4.3–4.9 ps (ref. 30 and 58) (Table S3 in the ESI†). No relevant twisting of the carotenoid chain occurs during the S_1_ → S_0_ decay in the gas phase.

Within OCP, the S_1_ → S_0_ relaxation dynamics is significantly different. First of all, the S_1_ population decay cannot be described by a single-exponential function, but at least two different decay times are required. This multiexponential decay character of S_1_ has been reported in several time-resolved spectroscopic studies of OCP.^5,14–16,36,59^ In particular, up to three different decay components were proposed to explain the S_1_ → S_0_ decay of keto-carotenoids bound to OCP, each attributed to a different species: (i) the “proper” dark S_1_ (pseudo 2A_g_^−^) state, (ii) an intramolecular charge transfer (ICT) state, and (iii) an additional dark state, called S*. The strongly coupled ICT and S_1_ states are generally associated with lifetimes of 0.5–0.9 ps and 2–5 ps,^5,14–16,36,59^ respectively, while a longer decay time (>7 ps) has been attributed to S*^15,16^ (Table S3 in the ESI†). There is still debate on the exact nature of the S* feature, although it is shared between several carotenoids.^29^ One explanation is that the S* feature arises from a “more twisted” conformation on the S_1_ potential energy surface. This distinct conformation could be the result of heterogeneity in the ground-state ensemble, or could arise within the excited-state dynamics by branching from S_2_.^29^

The experimental evidence of three different lifetimes is consistent with our analysis of the S_1_ sub-populations obtained from the puckering-based separation of the SH trajectories (Section 3.2). On the basis of our extracted decay times, we can associate the p^+^ and faster-decaying p^−^ trajectories with the S_1_/ICT state (0.8 ps and 1.2 ps lifetimes, corresponding to 52% and 14% of the S_1_ population, respectively), and the slower p^−^ component with S* (15 ps, 34%). This assignment also suggests a connection between the conformational distortion of CAN and the presence of the S* feature. Furthermore, the slower relaxation component can be easily associated with a ground-state conformational feature such as the puckering angle. For this reason, we attribute the appearance of an S* feature to the presence of more conformations of CAN within the ground-state ensemble. Surprisingly, our analysis of the DS dihedral suggests that S* is associated with a more planar structure (in the S_1_ state) than “proper” S_1_. Whether this observation could be specific to CAN in OCP, or more general to different carotenoids, has to be further investigated. According to our simulations, most of the relaxation process in OCP occurs through the S_1_/ICT state, while the slow-decaying S* component represents only a small portion of the S_1_ population, in agreement with experiments.^15,16^

Our identification of the S_1_ sub-populations supports the hypothesis that the multi-exponential character of S_1_ is due to the ground-state structural heterogeneity of the carotenoid in OCP.^14,36,55,60^ In fact, we could separate the two S_1_ sub-populations based only on the CAN conformation in the ground state. Conversely, no significant structural change was identified during the S_2_ → S_1_ relaxation. These results thus strongly support the ground-state heterogeneity origin of the S* feature, rather than a branching from a common S_2_ state. We could clearly associate the ground-state heterogeneity with the puckering conformation of the β_1_ ring of CAN, which in turn affects the dihedral angle between the π-conjugated chain and the β_1_ ring (DS, Fig. 4) in the ground state. The importance of the puckering of CAN keto-ring in the photoactivation mechanism of OCP has been previously suggested.^45,55^ Specifically, in a previous study by some of us,^45^ it has been shown that the potential of the keto-ring rotation depends on the puckering state. Moreover, Pishchalnikov *et al.*^55^ suggested that puckering can affect the position of the CAN keto-group in the CTD, and hence the hydrogen bond lengths between the carotenoid keto-oxygen and residues Y201 and W288.

### 4.2. Photoisomerization and photoactivation mechanism

For a fraction of SH trajectories (∼12%, most of which with p^+^ puckering, Table S2†), the twisting of DD at the S_1_ → S_0_ transition geometries leads to the *trans*-to-*cis* isomerization of C_7_=C_8_, accompanied by a partial torsion around C_6_–C_7_ (Fig. 5). To the best of our knowledge, this kind of photoisomerization, which we identified as a hula-twist mechanism,^56,61^ has not been reported yet for protein-bound carotenoids. Since this isomerization occurs in the CTD of OCP^O^, it represents a possible trigger for hydrogen bond rupture between the β_1_-ring of CAN and residues Y201 and W288 in OCP photoactivation. As a matter of fact, one proposed mechanism for hydrogen bond rupture involves the *trans*-to-*cis* s-photoisomerization of the C_6_–C_7_ single bond, which would flip the orientation of the β_1_ ring by 90°.^21^ The C_7_=C_8_ double bond isomerization observed in our SH simulations involves only a partial twisting of the adjacent C_6_–C_7_ single bond, which retains its s-*trans* conformation, and therefore does not lead to the 90° flip of the β_1_ ring (Fig. 5). This outcome is consistent with the spectroscopic investigation by Konold *et al.*,^15^ who excluded β_1_ ring rotation on the basis of pump-probe vis-IR absorption anisotropy measurements for OCP binding 3′-hydroxyechinenone (hECN). It should be noted that a complete flip of the β_1_ ring would be very unlikely in the tight OCP binding pocket, as it would be sterically hindered by the protein residues in close contact with the ring. In contrast, the photoisomerization mechanism observed in our simulations does not involve any large movement (only the movement of the methyl group connected to C_9_ is needed, see Fig. 5).

Recent time-resolved X-ray crystallography experiments^62^ revealed a concerted isomerization of two carotenoid single bonds located in the NTD, namely C_6′_–C_7′_ and C_8′_–C_9′_ (Fig. 1), through the so-called bicycle-pedal mechanism. This isomerization, occurring near the β_2_ ring, was proposed as a possible trigger for OCP photoactivation. In our simulations we do not observe this type of photoisomerization, as both the C_6′_–C_7′_ and C_8′_–C_9′_ single bonds preserve their initial conformations along all the SH trajectories. To resolve this discrepancy, we note that the first structural intermediate collected in time-resolved X-ray experiments corresponds to a 1 minute time delay after illumination. Notably, this time delay is much longer than the entire photoactivation time of OCP, and does not strictly represent the primary photoproduct, which is instead formed in the ps timescale.^15^ Furthermore, the experimental conditions employed in ref. 62 could have altered the photocycle of OCP due to the crystal packing. Finally, isomerization at the β_1_ ring seems a more likely cause of photoactivation, as (i) the β_1_ ring is conserved among different OCP-bound carotenoids, and (ii) it is the location of the H-bond disruption necessary for carotenoid translocation.

In our simulations, most of the photoisomerization events were observed in the early decaying trajectory group, *i.e.*, the one that we associate with the S_1_/ICT component of the S_1_ population, rather than S*. This is in agreement with a recently reported spectroscopic investigation,^16^ which ruled out the idea that S* is responsible for the formation of the primary photoproduct (P_1_) of OCP photoactivation^15^ and suggested instead that a small portion of the S_1_ population evolves into P_1_. In fact, if photoisomerization is the trigger for photoactivation of OCP, then the primary photoproduct would evolve from the short-lived components rather than from the long-lived one, according to our simulations. If we exclude from the analysis all trajectories that photoisomerize, the remaining p^+^ trajectories still present a short-lived S_1_ lifetime (on average 1.2 ps), indicating that photoisomerization is not the cause for the fast decay time, but rather that the two events are associated with the same structural features of CAN.

To conclude, photoisomerization at the C_7_=C_8_ double bond seems a likely trigger for the H-bond breaking that finally leads to CAN translocation. However, during our simulations we have not observed any breaking of the two hydrogen bonds with Y201 and W288. As the photoisomerization mechanism conserves the orientation of the β_1_ ring, there is no immediate force steering the CAN carbonyl away from the H-bonding residues. On the other hand, the new isomer state might lower the barrier towards H-bond dissociation and facilitate the translocation of CAN. The confirmation of this hypothesis however would require additional simulations over a longer time scale.

## 5. Conclusions

We have investigated the excited state dynamics of CAN in OCP by using QM/MM surface hopping nonadiabatic dynamics, analyzing both the ultrafast (200 fs) S_2_ → S_1_ internal conversion and the slower decay of S_1_ to the ground state. The ultrafast component of the dynamics presents a clear signature of the intermediate S_*X*_ state, which corresponds to the dark 1B_u_^−^ state, in agreement with our previous results on lutein in methanol solution.^37^ The similarities between the ultrafast dynamics of CAN in the gas phase and in OCP, and with lutein, suggest that a decay mediated by the S_*X*_ state may be common among carotenoids in different environments. Moving to the S_1_ state of CAN in OCP, a clear multiexponential decay has been found, with strikingly different lifetimes (∼1.2 ps and ∼15 ps), in agreement with spectroscopic measurements.^5,14–16,36,59^ On this basis, we have assigned the slowly decaying population to the so-called S* state. Analysis of the ground-state ensemble has also allowed us to unequivocally associate the S* population with a specific puckering conformation of the β_1_ ring, which is significantly twisted due to the hydrogen bonds to Y201 and W288. These results strongly support the ground-state heterogeneity model to explain the S* spectroscopic feature.^29^

Surprisingly, a small portion of SH trajectories showed isomerization of the C_7_=C_8_ double bond, through a space-conserving mechanism. As isomerization occurs exclusively on this double bond, in the CTD side of CAN, we propose this photochemical mechanism as a trigger for OCP photoactivation, possibly causing the rupture of the hydrogen bonds with Y201 and W288. Photoisomerization was strongly associated with the fast decaying portion of S_1_ population (*i.e.*, what we associate with the S_1_/ICT state, rather than S*), supporting S_1_/ICT as the species responsible for OCP photoactivation. Further simulations, covering much longer timescales, are needed to explain how isomerization can lead to the translocation of CAN into the NTD.

Our results reveal a fundamental role of the OCP binding pocket in tuning the photochemistry of CAN. The limited conformational freedom of CAN in OCP, combined with the constraints of hydrogen bonds and steric repulsion, are the keys to explain its specific photochemical response. In particular, the distortion generated by the hydrogen bonds on the β_1_ ring determines a selective isomerization at the C_7_=C_8_ double bond, which could be the trigger to OCP photoactivation.

## Data availability

The data underlying Fig. 2, 3, and 4 can be found on Zenodo at DOI: 10.5281/zenodo.8300809.

## Author contributions

BM and LC conceived this research. DA, LC, and BM developed the methodology. AA performed the simulations. AA, DA, and LC analyzed the results. BM and LC supervised the work. All authors wrote the manuscript.

## Conflicts of interest

There are no conflicts to declare.

## Supplementary Material

SC-014-D3SC02662K-s001
