# Supplementary material for: How orange carotenoid protein controls the excited state dynamics of canthaxanthin
Source: Chem Sci. 2023 Sep 22;14(40):11158–69. doi: 10.1039/d3sc02662k (PMC10583711; doi:10.1039/d3sc02662k)
Supplement: SC-014-D3SC02662K-s001 [file SC-014-D3SC02662K-s001.pdf]

Electronic Supplementary Material (ESI) for Chemical Science.
This journal is © The Royal Society of Chemistry 2023

Supporting Information: How Orange Carotenoid Protein controls the excited state dynamics of Canthaxanthin

Amanda Arcidiacono, Davide Accomasso, Lorenzo Cupellini, Benedetta Mennucci

Dipartimento di Chimica e Chimica Industriale, University of Pisa, Via G. Moruzzi 13, 56124 Pisa, Italy

S1 Selection of the active space of semiempirical FOMO-CISD method

The orbital active space and the floating occupation parameter w of the semiempirical FOMO-CISD method employed in the present work were selected by performing preliminary calculations for both canthaxanthin (CAN) and a keto-carotenoid model having the same π-conjugated chain of CAN. The calculations for the model were performed to have a comparison with reference data obtained with high level *ab initio* calculations, feasible for highly symmetric systems, in a previous work of the group [1]. In this benchmark the excitation energies were computed at the GS minimum geometry in a Frank-Condon (FC) framework. The reference is reported next to the semiempirical calculations with different active spaces in Table S1A. For these calculations, the geometry of the model was optimized at the MP2 level since it can provide a sufficiently correct treatment of dynamic correlation, which is necessary for long conjugated chains to recover the correct degree of delocalization. The purpose of these calculations was to select a well suited active space to be used as a reference for the following calculations on CAN (for which it is not possible to have a reliable *ab initio* treatment). We therefore chose the orbital active space (14,12) as reference since it manages to treat at the same time in a sufficiently accurate way both the $2A_g^-$ and the $1B_u^+$ vertical energies, also including the n orbitals and allowing to compute the $n \rightarrow \pi^*$ states (A_u and B_g).

As done for the model system, we computed the excitation energies of CAN, both at S_0 and S_1 minimum geometries, this time optimized at the semiempirical level (Table S1B). We could verify

that the active space (14,12) previously chosen for the model and a smaller active space of (8,10) gave very similar results, which were also consistent with some experimental values from the literature for the vertical excitation energy of $1B_u^+$ [2], and the adiabatic energy of $2A_g^-$ [3]. We therefore decided to adopt an active space of (8,10) in the simulations to further reduce the computational cost and because the $n \to \pi^*$ states are not likely to be relevant in the excited state process.

Table S1: (A) Comparison between the Benchmark FC excitation energies (in eV) and the R-AM1/FOMO-CISD FC excitation energies (in eV) computed with different combinations of the AS and the w parameter. The GS minimum geometry is in every case obtained with MP2. Some of the ASs selected contain n type orbitals, therefore the states A_u and B_g can be computed. (B) Excitation energies (in eV) for CAN's low-lying excited states computed at the R-AM1/FOMO-CISD level using the active spaces (14,12) and (8,10), and a Gaussian width (w) of 0.2 and 0.1 Hartree, respectively. On the left are listed the Frank-Condon excitation energies computed at the S_0 minimum geometry, while on the right are displayed the excitation energies at S_1 minimum geometry, and in both cases the state energies are reported relative to the S_0 energy at S_0 minimum geometry. All the geometries were optimized with the same R-AM1/FOMO-CISD method used for the calculation of the state energies. (C) The experimental data for the FC energy of $1B_u^+$ and the adiabatic energy of $2A_g^-$ are reported.

(A) *model system data*

State	Benchmark	$(8,8)$ $w = 0.2h$	$(8,10)$ $w = 0.1h$	$(12,10)$ $w = 0.1h$	$(14,12)$ $w = 0.2h$
S_0 (A_g)	0.00	0.00	0.00	0.00	0.00
S_1 (A_g)	2.54	2.53	2.64	2.63	2.61
S_2 (B_u^+)	2.93	2.82	2.90	2.80	2.83
S_3 (B_u^-)	3.21	3.05	3.08	3.12	3.19
S_4 (A_u)	3.57	-	-	-	3.60
S_5 (B_g)	3.57	-	-	-	3.60

(B) *CAN data*

State S_0 min. geom.	$(14,12)$ $w = 0.2h$	$(8,10)$ $w = 0.1h$	State S_1 min. geom.	$(14,12)$ $w = 0.2h$	$(8,10)$ $w = 0.1h$
S_0 $(1^1A_g^-)^*$	0.00	0.00	S_0 $(1^1A_g^-)$	0.42	0.43
S_1 $(2^1A_g^-)$	2.53	2.50	S_1 $(2^1A_g^-)^*$	1.97	1.89
S_2 $(1^1B_u^+)$	2.77	2.70	S_2 $(1^1B_u^-)$	2.72	2.63
S_3 $(1^1B_u^-)$	3.07	3.03	S_3 $(1^1B_u^+)$	2.77	2.70
S_4 (1^1B_g)	3.49	-	S_4 $(3^1A_g^-)$	3.39	3.17
S_5 (1^1A_u)	3.49	-	S_5 (1^1B_g)	3.64	-
S_6 $(3^1A_g^-)$	3.71	3.48	S_6 (1^1A_u)	3.64	-

(C) *CAN experimental data*

$1^1B_u^+$	Supercritical CO_2 2.74	Hexane 2.68	$2^1A_g^-$	1.65

S2 Thermal equilibrations in the ground state

An ensemble of nuclear coordinates and velocities of CAN was generated by running thermal equilibrations in the ground state (S_0). For the simulations of CAN in OCP, we performed ten QM/MM thermal equilibrations, propagated for 10 ps using the Bussi-Parrinello thermostat [4], with a relaxation time constant of 10 fs. The starting coordinates for the QM/MM thermal equilibrations were extrated from a classical molecular dynamics trajectory of OCP, previously computed in our group [5]. For CAN in the gas phase, one thermal equilibration was propagated for 25 ps using the van Gunsteren-Berendsen thermostat [6], with a friction coefficient of 5.0×10^{13} s^{-1} for all the atoms. In all the thermal equilibrations, we used a temperature of 300 K and a time step of 0.5 fs. In the sampling of initial conditions for the surface hopping simulations, we used the last 5 ps of the ten QM/MM thermal equilibrations of CAN in OCP, and the last 20 ps of the gas phase thermalization.

The data on the thermal equilibrations is reported below.

S2.1 Gas phase

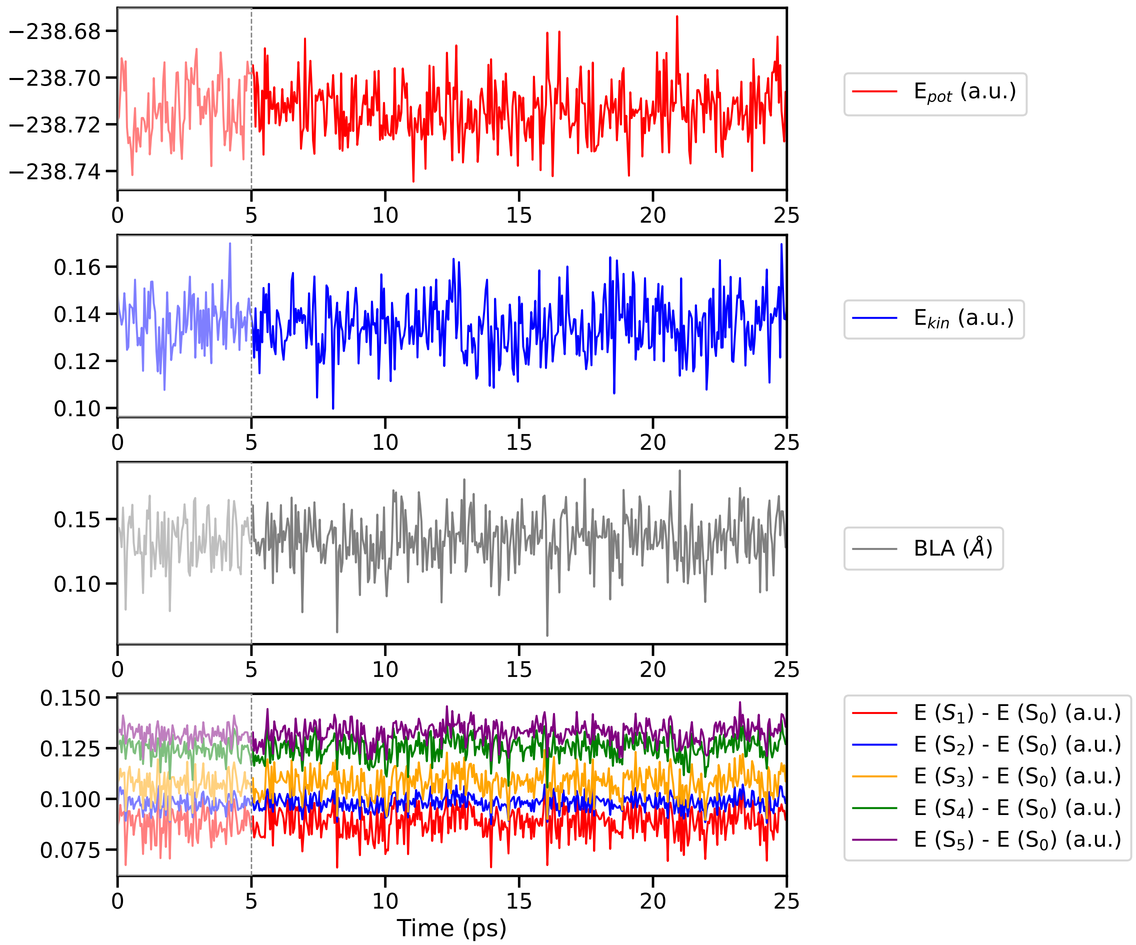

Figure S1: Data on the thermal equilibration trajectory of CAN in vacuum. From top to bottom: potential energy (Hartree), kinetic energy (Hartree), bond length alternation (BLA) coordinate value (angstrom), excitation energies of the first 5 low-lying excited states (Hartree). The dimmed section is the discarded non-equilibrium portion of the trajectory, the rest was used for the sampling of the SH initial conditions.

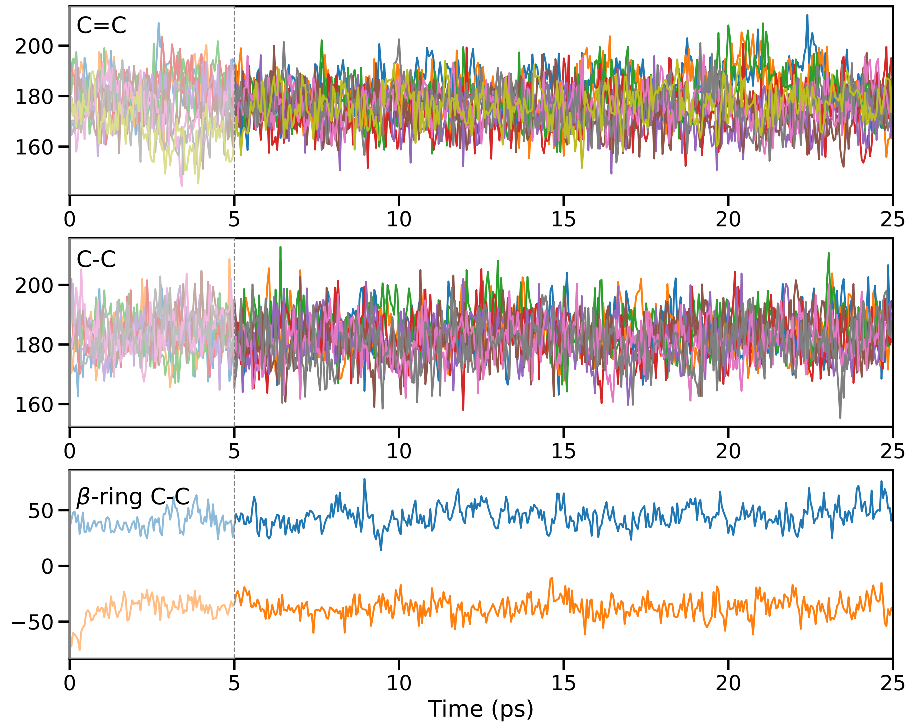

Figure S2: Values of the chain's dihedral angles (degrees) during the thermal equilibration of CAN in vacuum. From top to bottom: C=C dihedrals, internal C-C dihedrals, β-ring connection terminal C-C dihedrals. The dimmed section is the discarded non-equilibrium portion of the trajectory, the rest was used for the sampling of the SH initial conditions.

S2.2 OCP

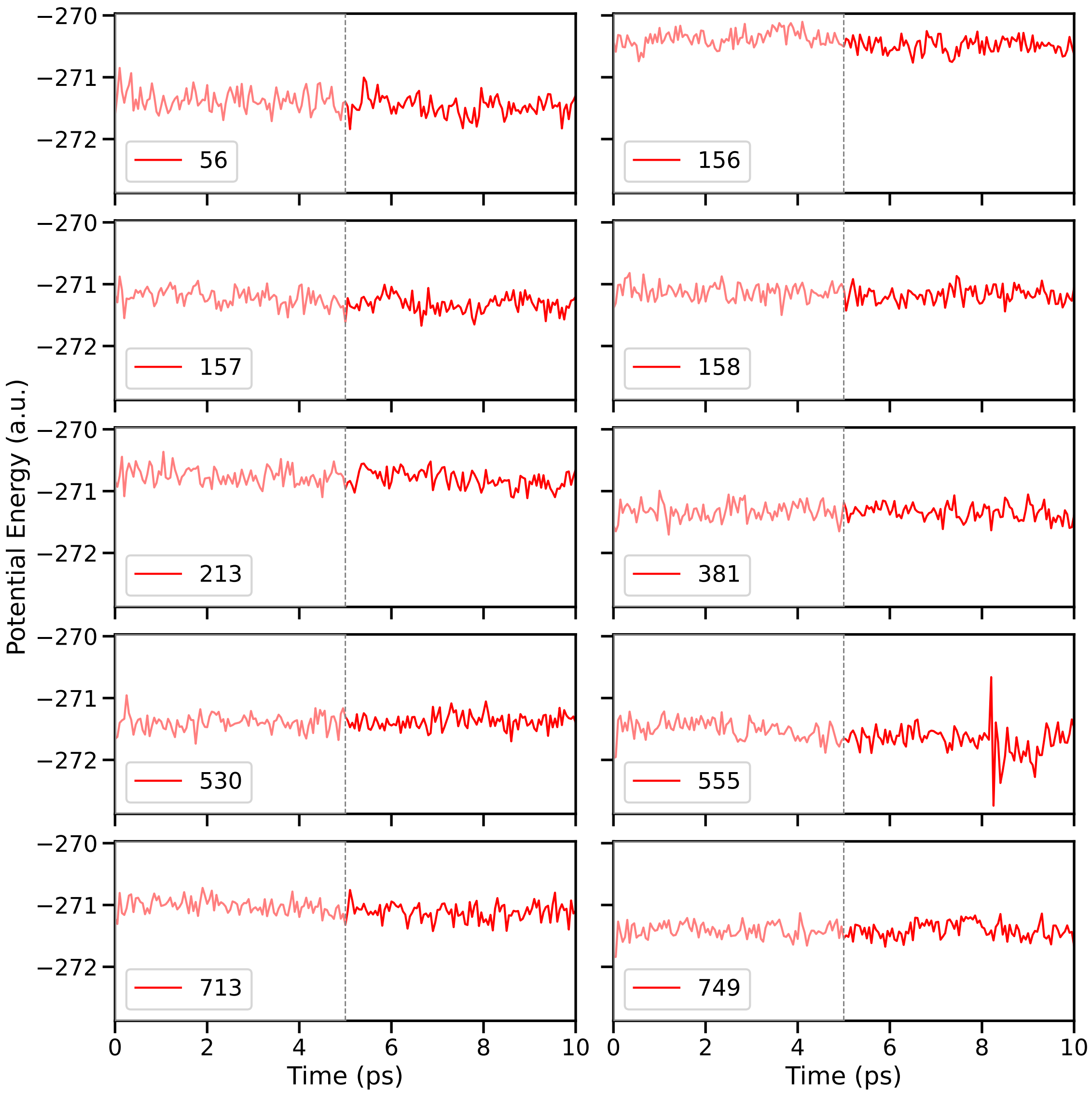

Figure S3: Potential energy (Hartree) during the 10 thermal equilibration trajectories of CAN in OCP. The numbers refer to the frames smpled from a classical MD trajectory [5].

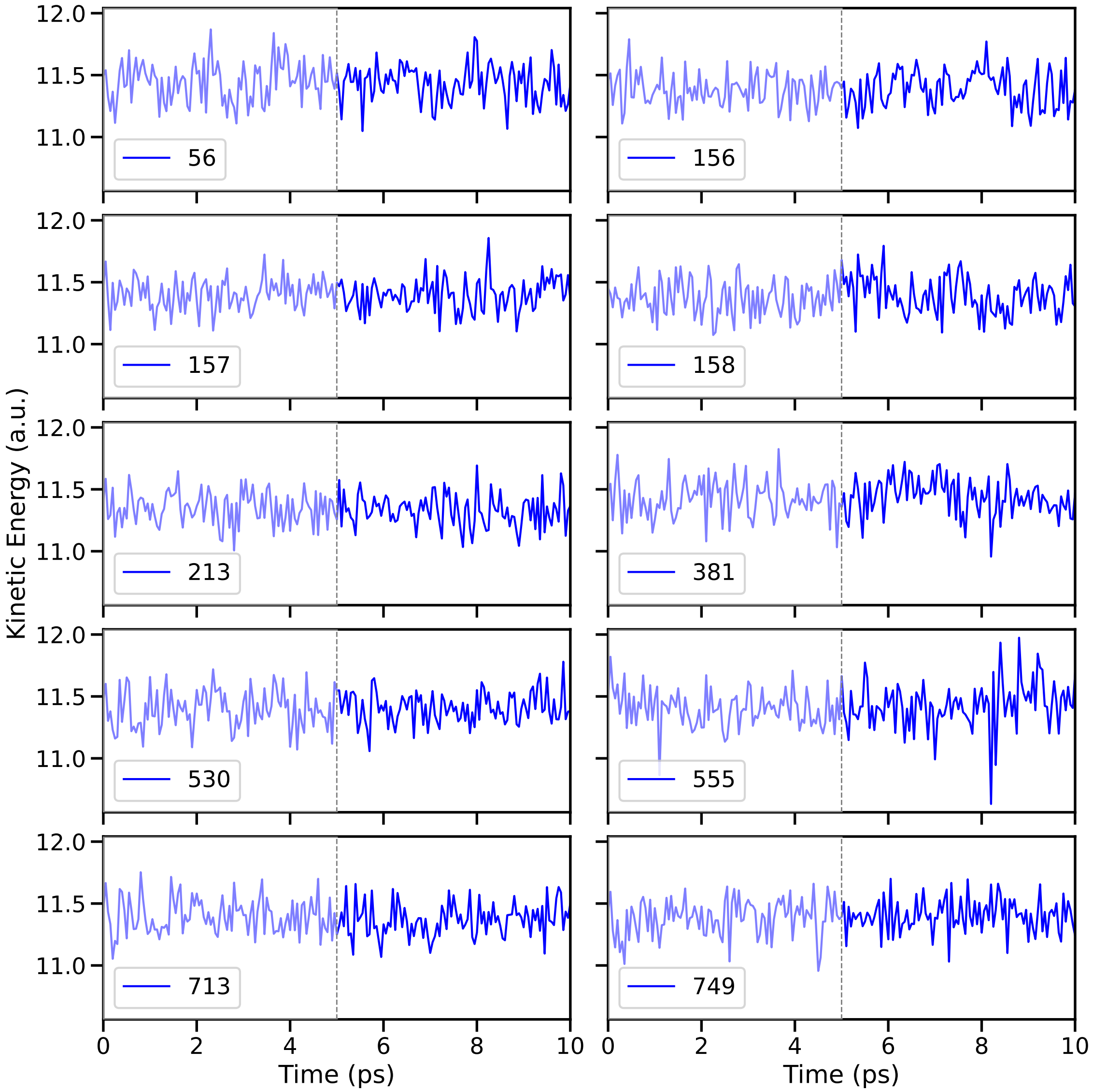

Figure S4: Kinetic energy (Hartree) during the 10 thermal equilibration trajectories of CAN in OCP. The numbers refer to the frames smpled from a classical MD trajectory [5].

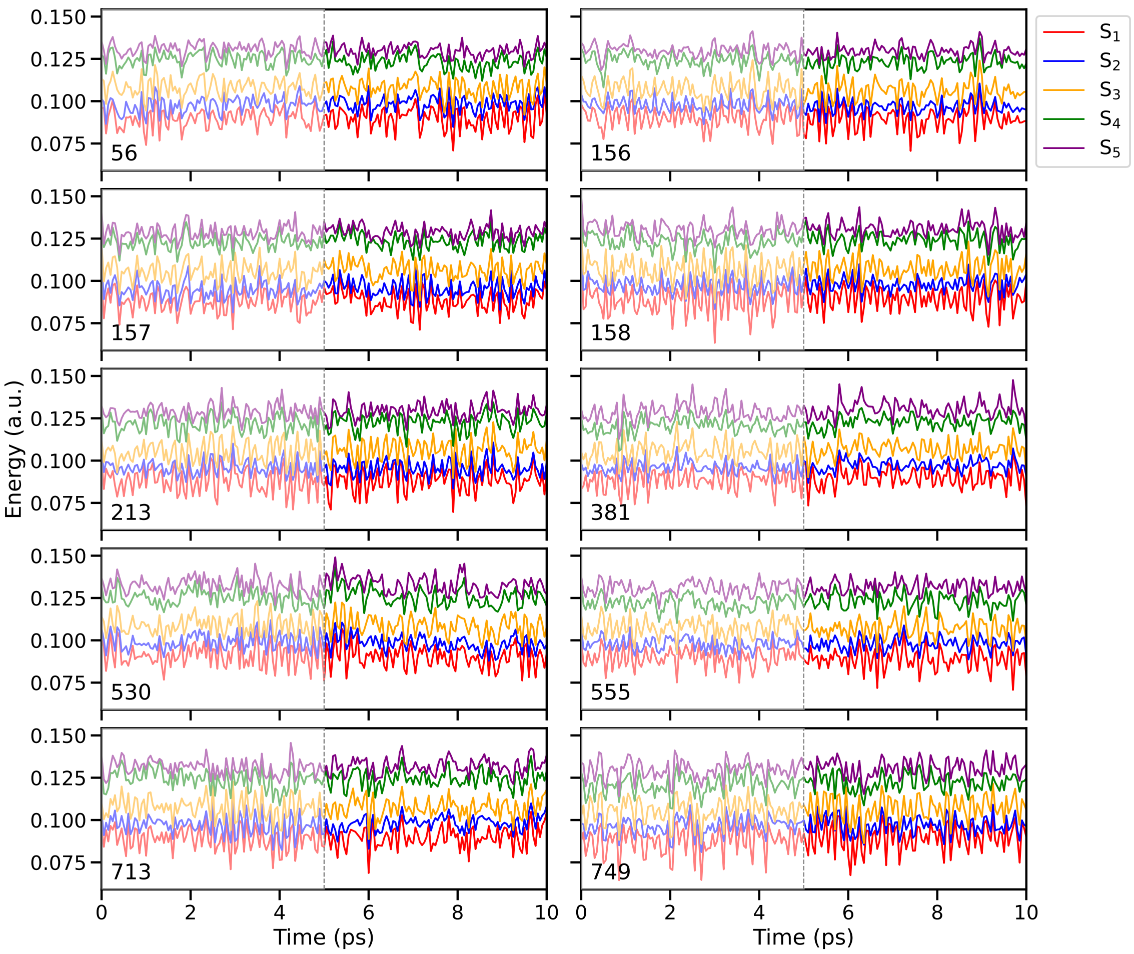

Figure S5: Excitation energies (Hartree) of the first 5 low-lying excited states of CAN during the 10 thermal equilibration trajectories of CAN in OCP. The numbers refer to the frames sampled from a classical MD trajectory [5].

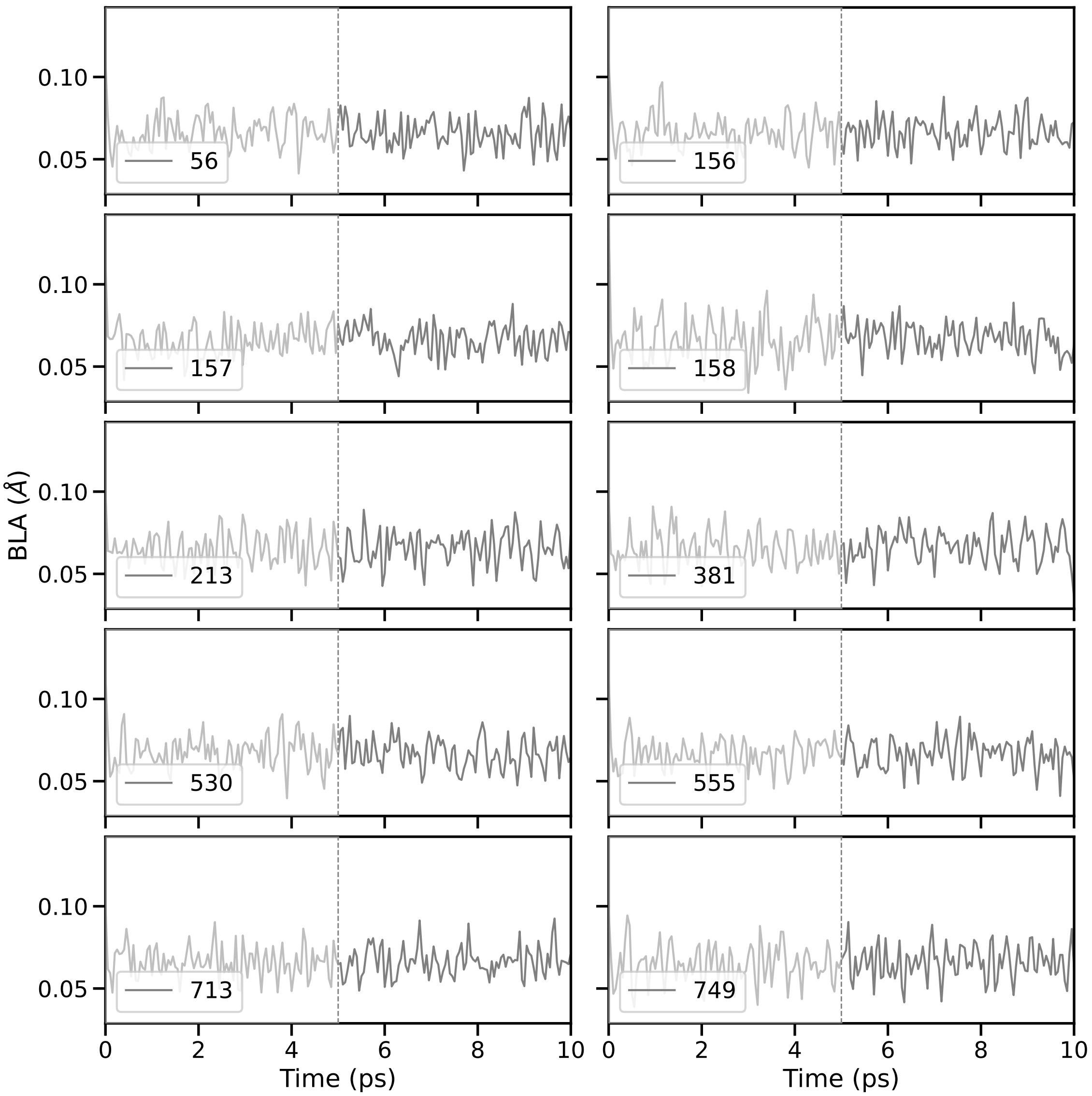

Figure S6: BLA value (angstrom) of CAN during the 10 thermal equilibrations of CAN in OCP. The numbers refer to the frames sampled from a classical MD trajectory [5].

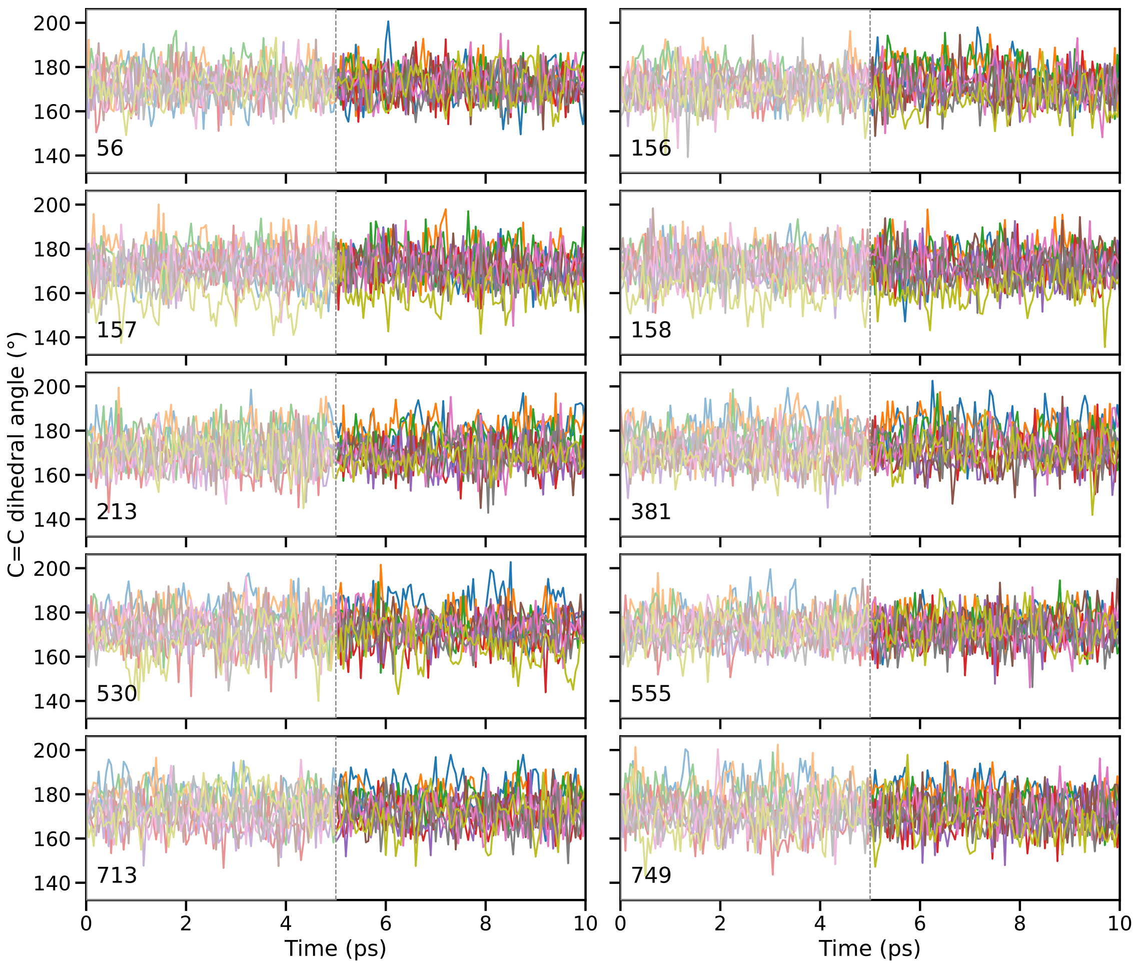

Figure S7: Values of the C=C dihedral angles (degrees) of CAN during the 10 thermal equilibrations of CAN in OCP. The numbers refer to the frames sampled from a classical MD trajectory [5].

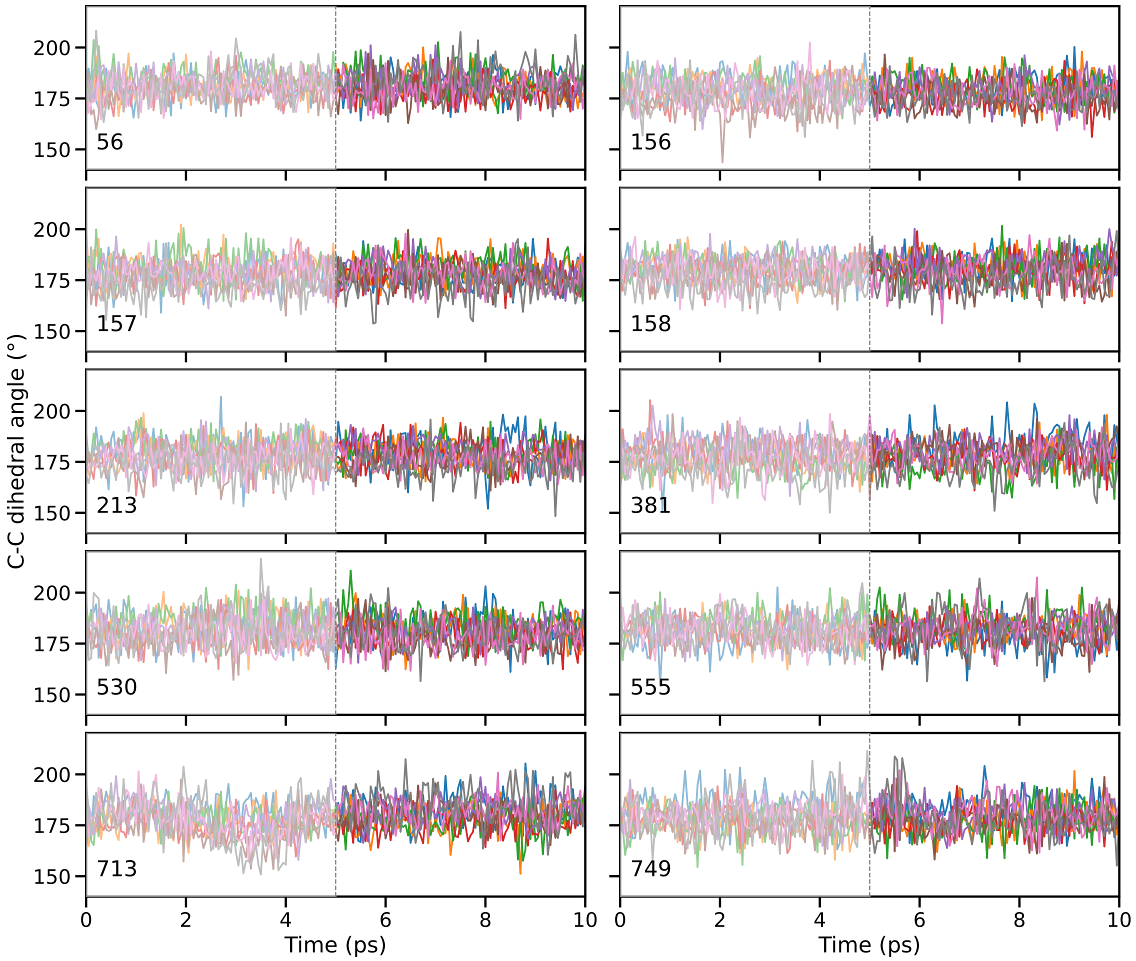

Figure S8: Values of the C-C dihedral angles (degrees) of CAN during the 10 thermal equilibrations of CAN in OCP. The numbers refer to the frames sampled from a classical MD trajectory [5].

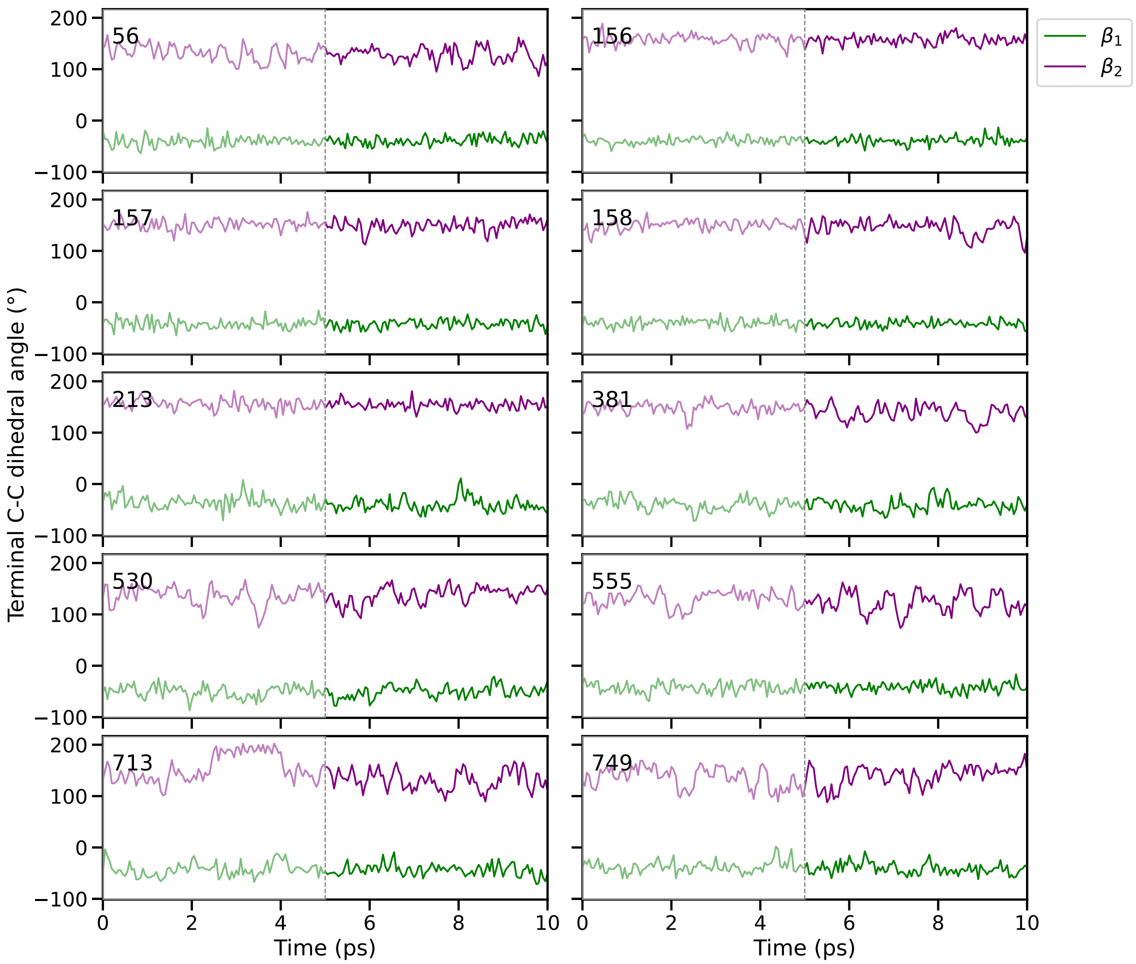

Figure S9: Values of the terminal C-C dihedral angles (degrees) of CAN during the 10 thermal equilibrations of CAN in OCP. The numbers refer to the frames sampled from a classical MD trajectory [5].

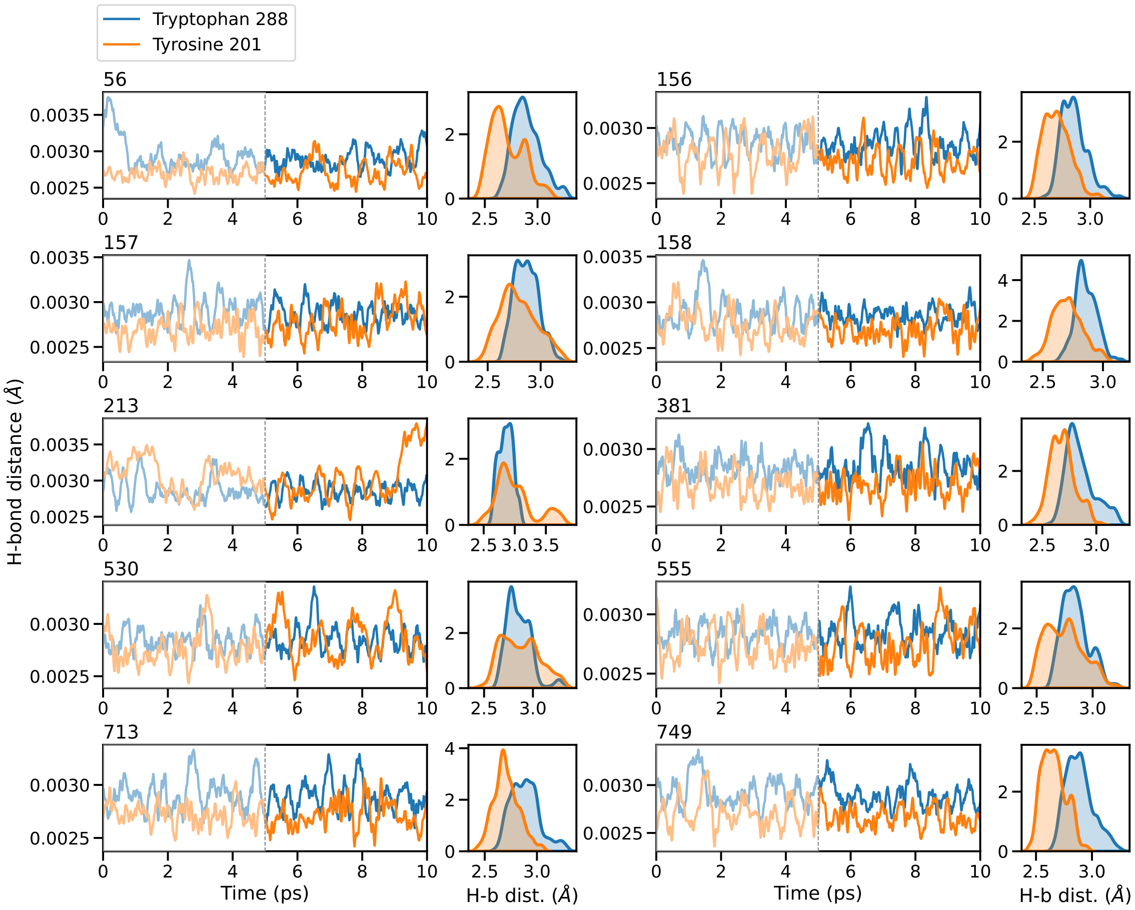

Figure S10: H-bond lengths (angstrom) of CAN with TYR201 (orange) and TRP288 (blue) during the 10 thermal equilibrations of CAN in OCP. We report both the the plots and the distributions. The numbers refer to the frames sampled from a classical MD trajectory [5].

S3 Diabatization

A diabatic wavefunction [7, 8, 9, 10] can be defined as a linear combination of adiabatic states and determine a unitary transformation from the adiabatic basis to the diabatic one:

$$|\boldsymbol{\eta}\rangle = |\boldsymbol{\varphi}\rangle \, \mathbf{T} \tag{S1}$$

with $|\boldsymbol{\eta}\rangle$ is the vector of the diabatic basis functions and $|\boldsymbol{\varphi}\rangle$ is the one of the adiabatic ones. $\mathbf{T}$ is a unitary rotation matrix built using some arbitrarily chosen reference states $|\boldsymbol{R}\rangle$ that must have a well defined character (ionic or neutral, localized or charge transfer excitation, etc.). By definition, a diabatic basis annihilates the derivative couplings, therefore it allows the preservation of the physical character and symmetry of each state near strong-coupling regions.
Instead of constructing a diabatic basis where the non-adiabatic couplings vanish completely (the so-called *crude* diabatic basis), it is possible to find a unitary matrix $\mathbf{T}$ transforming a truncated adiabatic basis into a strictly diabatic one only for one internal coordinate at a time or along a given path, and to obtain a basis where the couplings are not completely cancelled but they can become negligibly small. We use a diabatization technique where the reference states $\boldsymbol{R}$ chosen to build the rotation matrix $\mathbf{T}$ are the adiabatic states at S_0 minimum geometry:

$$|\boldsymbol{R}\rangle = |\boldsymbol{\psi}\rangle_{\boldsymbol{Q}=\boldsymbol{Q}_{S_0^{min}}} \tag{S2}$$

$\mathbf{T}$ is defined so that the sum of the overlaps between the diabatic wave functions and the reference is maximized, so that the the obtained diabatic functions are as similar as possible to the references. This is done by projecting the reference states onto the space spanned by the adiabatic states and then applying a Löwdin orthogonalization [11]:

$$T_{kj} = \sum_{l=1}^{N} \langle \varphi_k \rangle \, R_l (S^{-1/2})_{lj} \tag{S3}$$

$\boldsymbol{S}$ is the overlap matrix between the projections of the reference states onto the adiabatic basis:

$$|\bar{\eta}_l\rangle = \sum_{k=1}^{N} |\varphi_k\rangle \langle \varphi_k \rangle \, R_l \tag{S4}$$

$$S_{lj} = \langle \bar{\eta}_l \rangle \, \bar{\eta}_j \tag{S5}$$

The diabatic energies are the diagonal elements of the Hamiltonian matrix in the diabatic repre-

sentation (which does not diagonalize the Hamiltonian, unlike the adiabatic basis):

$$\begin{aligned} \mathbf{H} &= \langle \boldsymbol{\eta} | \mathcal{H}_{el} | \boldsymbol{\eta} \rangle = \\ &= \mathbf{T}^{\dagger} \langle \boldsymbol{\varphi} | \mathcal{H}_{el} | \boldsymbol{\varphi} \rangle \, \mathbf{T} = \\ &= \mathbf{T}^{\dagger} \mathbf{E} \mathbf{T}. \end{aligned} \tag{S6}$$

S4 Diabatic analysis of the ultrafast process

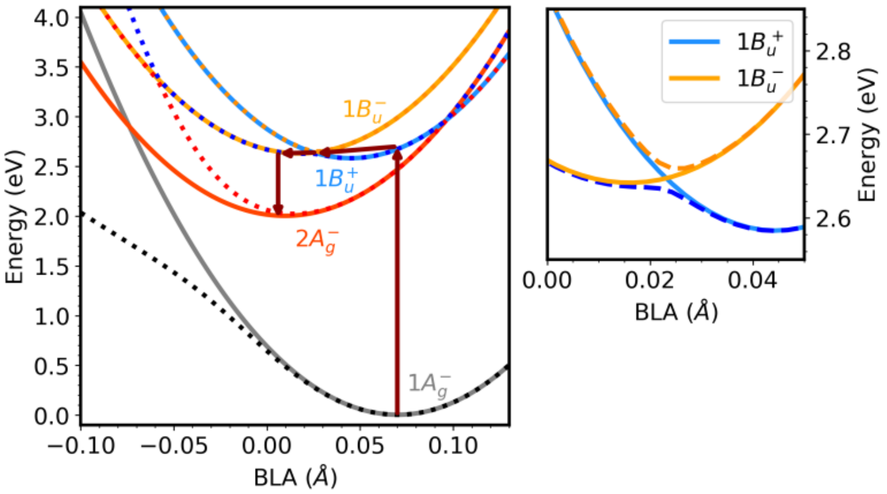

Figure S11: Diabatic (whole lines) and adiabatic (dashed lines) potential energy curves for the first four low-lying states of CAN in vacuo. The plotted lines are second-degree polynomial function fittings of the data obtained from the relaxed scan along the BLA computed at the R-AM1/FOMO-CISD level. The arrows highlight the path followed by the electronic population. On the right there is a focused image of the avoided crossing between S_2 and S_3 (dashed lines); the solid lines are the diabatic states $1B_u^+$ and $1B_u^-$.

To understand the nature of the excited states we adopt a quasi-diabatic representation (from now on, diabatic), defined on the basis of the overlap to reference states [7, 10]. Here the reference states of CAN were calculated at the S_0 minimum geometry. Figure S11 shows the diabatic states along the bond-length-alternation (BLA) coordinate with their adiabatic counterpart, painting a picture analogous to what found for Lutein [12]. BLA is defined by the following equation:

$$BLA = \frac{1}{N_s} \sum_{i=1}^{N_s} d_s^{(i)} - \frac{1}{N_d} \sum_{i=1}^{N_d} d_d^{(i)} \tag{S7}$$

where N_s and N_d are respectively the number of single and double bonds and d_s and d_d are their respective lengths. At the Franck-Condon point, states S_1–S_3 correspond to $2A_g^-$, $1B_u^+$, and $1B_u^-$, respectively. Vertical excitation populates $1B_u^+$ (S_2), and then CAN rearranges its geometry towards smaller BLA values, following the excited-state gradient. The nuclear wavepacket oscillates back and forth along the BLA coordinate, encountering the crossing region between $1B_u^+$ and $1B_u^-$ (See Figure S12). Passing through the crossing, the nuclear wavepacket can either remain in the $1B_u^+$ state or switch to $1B_u^-$. From an adiabatic point of view, in the former case the population of S_3 increases when the BLA reaches smaller values, while in the latter the population remains on S_2. Notably, the one-dimensional picture of Figure S11 shows a higher energy for the $1B_u^-$ minimum than $1B_u^+$, but along the dynamics other degrees of freedom are activated, lowering the energy of the $1B_u^-$ state until its population becomes prevalent. After ∼100 fs, the S_2 state has a prevalent $1B_u^-$ character, and the population oscillations stop as $1B_u^+$ is not reachable anymore. This is confirmed

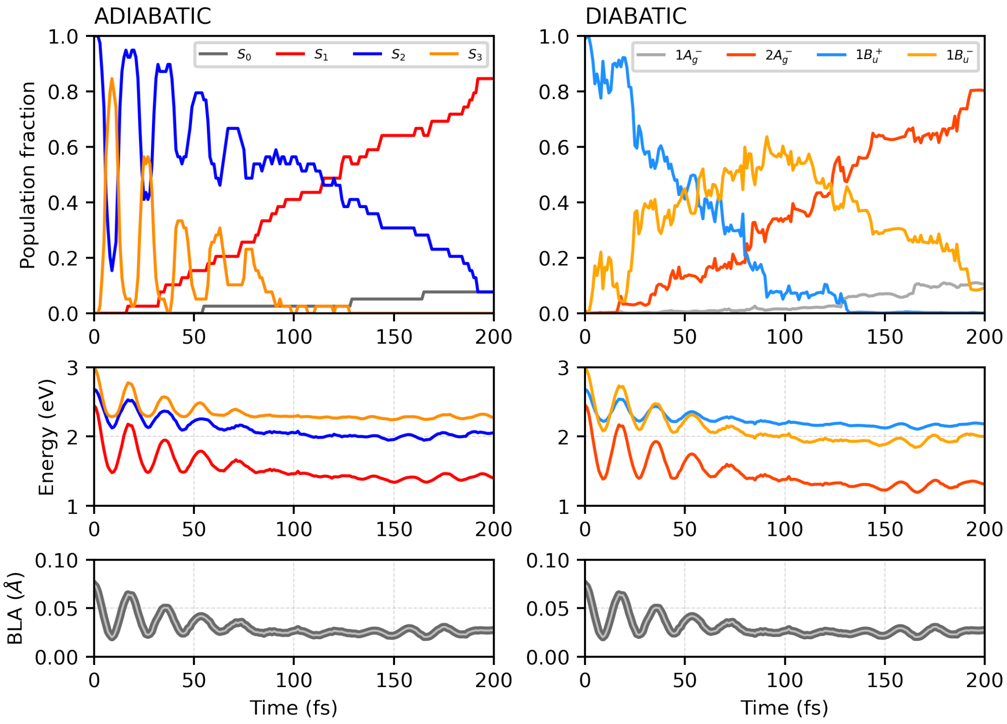

Figure S12: Ultrafast portion of CAN excited-state dynamics in vacuo. Results are obtained in the same way discussed in the main text. The panels on the left show the adiabatic state populations of CAN during the first 200 fs of the SH simulations. Below are reported, respectively, the excitation energies from the ground state and the BLA values. On the right, the populations and the energies are shown in their diabatic representation, obtained through the diabatizaton discussed in Section S3.

by looking at the populations of the diabatic states (Figure S12) along the gas-phase trajectories. This point occurs earlier in the OCP dynamics, as the energies of the two states separate earlier. This is likely due to the fact that more degrees of freedom are present within OCP than in vacuum.

S5 The $S_1 \rightarrow S_0$ relaxation process

The S_1 population of CAN in gas phase was fitted using the function:

$$P_1(t) = P_1(0)e^{-t/\tau_{21}} + P_2(0)\frac{\tau_{21}}{\tau_{21}+\tau_{10}}(e^{-t/\tau_{21}} - e^{-t/\tau_{10}}) \tag{S8}$$

where τ_{10} and τ_{21} are the lifetimes of S_1 and S_2+S_3, respectively, and $P_1(0)$ and $P_2(0)$ are the corresponding initial populations. In the gas phase case, $P_1(0) = 0$ and $P_2(0) = 1$, since all SH trajectories start from S_2.

The S_1 population of CAN in OCP was fitted using this bi-exponential function:

$$\begin{aligned} P_1(t) = w(P_1(0)e^{-t/\tau_{21}} + P_2(0)\frac{\tau_2 1}{\tau_{21}+\tau_A}(e^{-t/\tau_{21}} - e^{-t/\tau_A}))+ \\ + (1-w)(P_1(0)e^{-t/\tau_{21}} + P_2(0)\frac{\tau_{21}}{\tau_{21}+\tau_B}(e^{-t/\tau_{21}} - e^{-t/\tau_B})) \end{aligned} \tag{S9}$$

where τ_{21} is the lifetime of S_2+S_3 and τ_A and τ_B are the two lifetimes of S_1. $w_A = w$ and $w_B = 1-w$ are the two weights assigned to τ_A and τ_B. In this case the initial populations are $P_1(0) = 0.11$ and $P_2(0) = 0.89$.

S6 Puckering

The *puckering* of a six-term ring was described by Cremer and Pople [13] with two coordinates, θ and ϕ, which map all the possible conformations of such ring in a polar coordinate system. In the case of CAN's terminal rings, that contain a double bond and a carbonyl group, the degrees of freedom of the possible conformations assumed by a normal six-term ring are reduced, θ ad ϕ are perfectly correlated (Figure S13) and the rings can only assume two half-boat conformations (Figure S14). We therefore only use the ϕ coordinate to describe the ring's puckering.

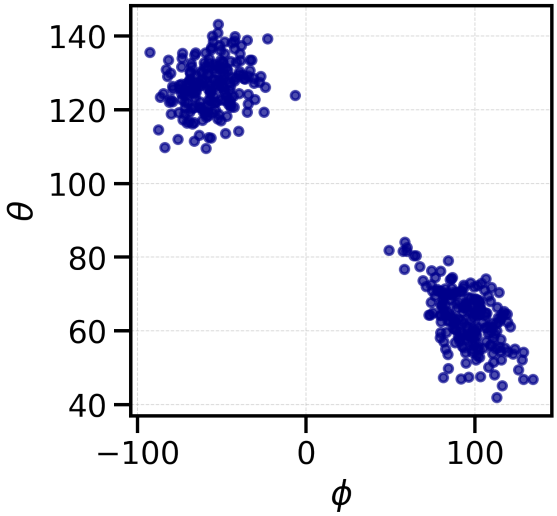

Figure S13: Correlation between the Cremer and Pople's θ and ϕ coordinate in the case of CAN's β_1 ring, a six-term ring with three sp2 carbons.

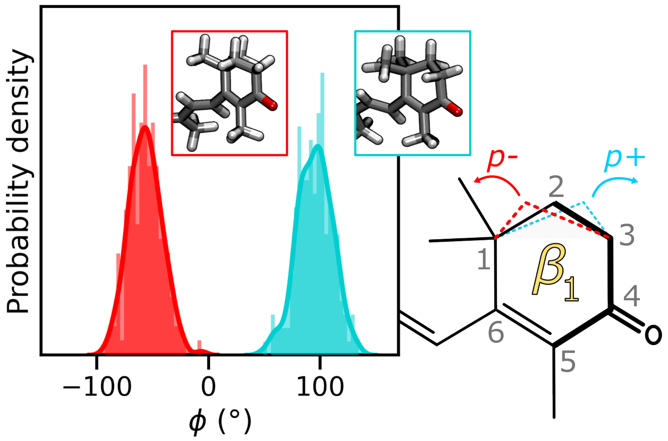

Figure S14: Two representative structures and distribution of the puckering coordinate (ϕ) of the β_1 ring in the initial conditions of the SH trajectories. Structures with $\phi < 0$ are associated with the p^- conformation, while the ones with $\phi \geq 0$ define the p^+ conformation.

S7 Supporting figures

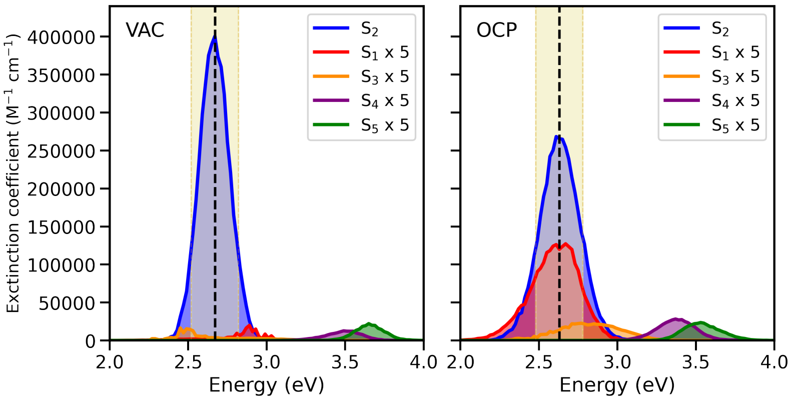

Figure S15: Absorption spectrum of CAN in vacuum (left) and in OCP (right). Every plotted value except the ones relative to S_2 (the blue line) is multiplied by a factor of 5.

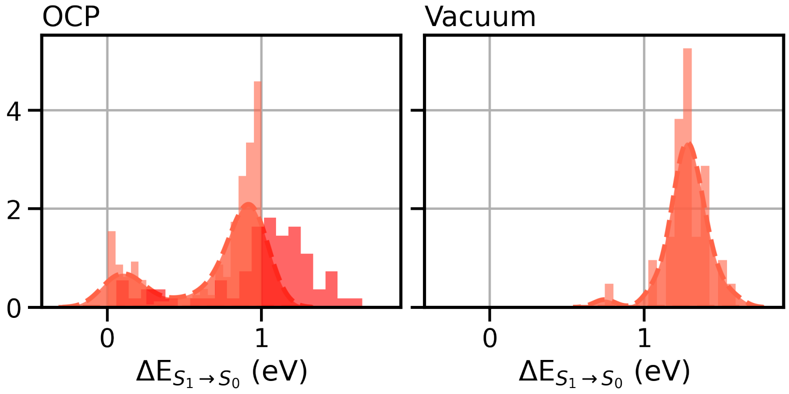

Figure S16: Distribution of the ΔE value (eV) between S_1 and S_0 at $S_1 \rightarrow S_0$ hopping times of the surface hopping trajectories. On the left we show the simulations in OCP, the distribution in red corresponds to a sample of trajectories that were ran with no constraint on the energy gap for allowing the hops (i.e. the hops are allowed only if the energy difference between the involved states is $\leq$ than the imposed threshold), while in orange such threshold is of 1.0 eV. On the right we show the simulations in vacuum, where no constrained were applied.

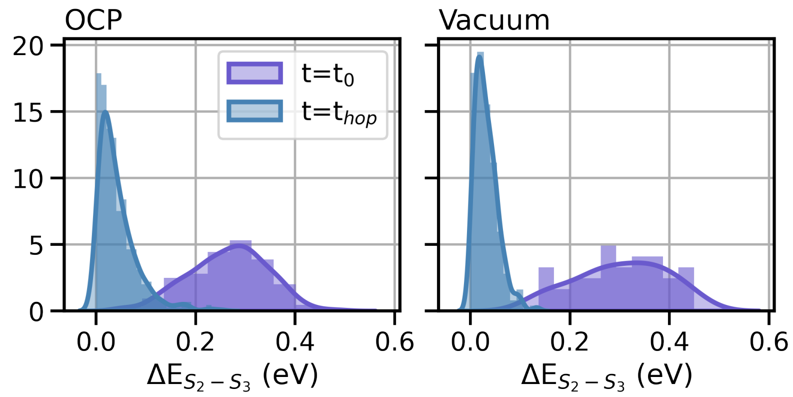

Figure S17: Distribution of the ΔE absolute value (eV) between S_2 and S_3. The purple distribution is referred to the ground state initial conditions of the surface hopping trajectories, the blue distribution is the energy gap between S_2 and S_3 at $S_2 \rightarrow S_3$ and $S_3 \rightarrow S_2$ hopping times. On the left we show the data from the simulations in OCP, on the right from the simulations in vacuum.

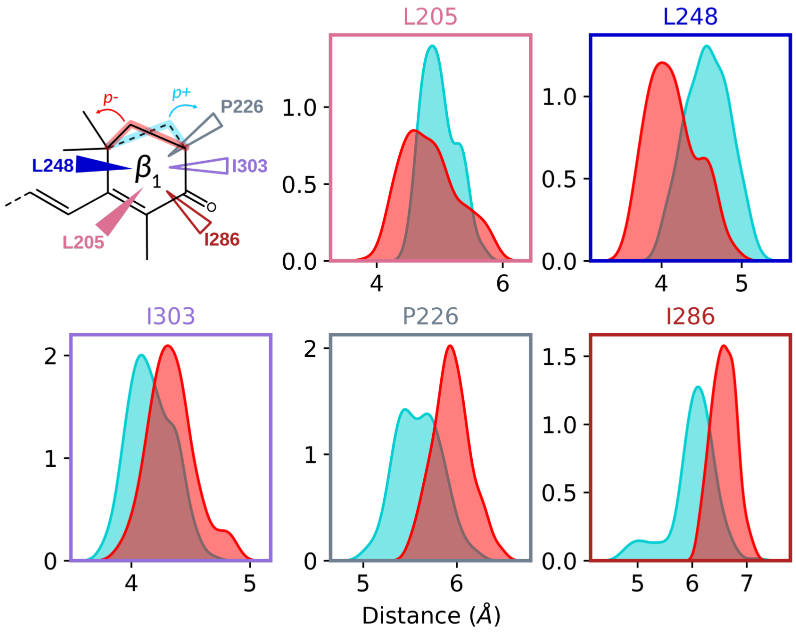

Figure S18: Correlation between the puckering conformation and the distributions of the distances of 5 CTD residues.

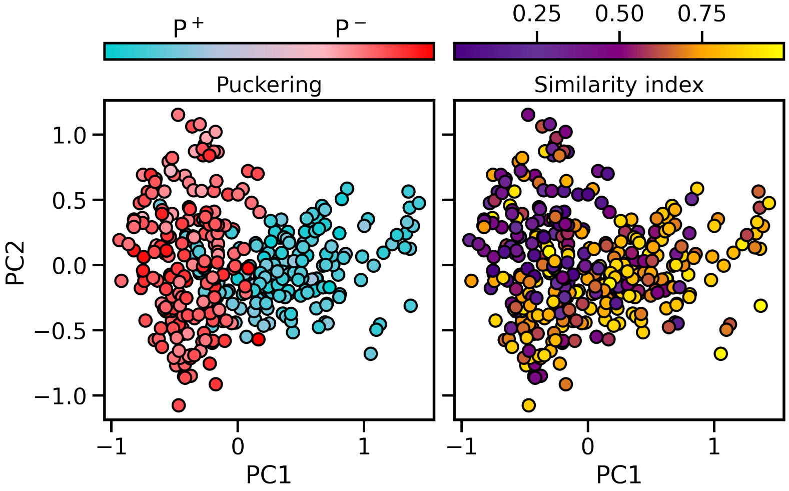

Figure S19: Left panel: scatter plot of the PCA observations highlighting the value of ϕ (puckering angle) of each geometry with the red/light-blue color gradient (p$^-$ in red and p$^+$ in light-blue). Right panel: the same plot of the PCA observations in which is reported with the purple/yellow color gradient the value of s_{DS} (purple corresponds to more distorted DS and yellow to more flat DS).

Table S2: Average values of several relevant properties for each group obtained from the puckering-based separation. From top to bottom, lifetime of the S_1 excited state (fitting according to Equations S8 and S9 for $\mathbf{p}^+$ and $\mathbf{p}^-$, respectively), similarity index computed for the dihedral DS in the initial conditions, similarity index computed for the dihedral DD at $S_1 \rightarrow S_0$ hopping times, energy gap between S_1 and S_0 at hops and percentage of trajectories that undergo the hula-twist isomerization during the $S_1 \rightarrow S_0$ decay.

**Property**	$\mathbf{p}^+$	$\mathbf{p}^-$
S_1 lifetime(s)	0.84 (ps)	1.21, 15.3 (ps)
Similarity index of DS at $t = 0$	0.51	0.73
Similarity index of DD at $t = t_{hop}$	0.33	0.54
Energy gap at $S_1 \rightarrow S_0$ hops	0.64 (eV)	1.06 (eV)
Isomerization yield	18.2 %	4.79 %

Table S3: State lifetimes (in ps) for keto-carotenoids canthaxanthin (CAN), echinenone (ECN), and 3-hydroxyl-echinenone (hECN), determined by time-resolved spectroscopy in different solvents and proteins (i.e. the orange carotenoid protein, OCP, and the red carotenoid protein, RCP).

Carotenoid	Environment	State lifetime (ps) S_2	S_1	ICT	S^*	Technique	Reference
CAN	benzene	-	4.5	-	9.1	transient abs.	Chabera 2009 [14]
	DMSO	-	4.9	-	9.1	transient abs.	Chabera 2009 [14]
	hexane	0.1	4.3	-	-	transient abs.	Khan 2020 [15]
	methanol	0.08	4.7	-	-	transient abs.	Khan 2020 [15]
	OCP (12 °C)	∼0.1	3.1-3.4, 6.1-7.4 [a, b]	-	-	transient abs.	Slouf 2017 [16]
	RCP (12 °C)	<0.1	3.6	0.4	-	transient abs.	Slouf 2017 [16]
ECN	benzene	-	6.2	-	-	transient abs.	Chabera 2009 [14]
	DMSO	-	6.4	-	-	transient abs.	Chabera 2009 [14]
	OCP^O	0.06	3.8 [e]	0.9 [e]	-	transient abs.	Berera 2012 [17]
	OCP^R	0.06	3.2 [e]	0.6 [e]	-	transient abs.	Berera 2012 [17]
	OCP (12 °C)	∼0.1	2.4-3.3 [b]	0.5-0.9 [b]	-	transient abs.	Slouf 2017 [16]
	OCP (22 °C)	0.08 ps	2.4	0.58	7.3	transient abs.	Nizinski 2022 [18]
hECN	CS_2	0.135	6.8	-	-	transient abs.	Polivka 2005 [19]
	n-hexane	0.230	6.4	-	-	transient abs.	Polivka 2005 [19]
	methanol	0.185	6.2	-	-	transient abs.	Polivka 2005 [19]
	OCP	<0.1	0.9, 3.3 [c]		-	transient abs.	Polivka 2005 [19]
	OCP	0.1	1	4.5	-	transient abs.	Wilson 2008 [20]
	OCP^O (77 K)	0.34	7.5 [e]	2.0 [e]	-	transient abs.	Berera 2013 [21]
	OCP^R (77 K)	0.26	7.6 [e]	2.3 [e]	-	transient abs.	Berera 2013 [21]
	OCP	≈0.1	1.4, 4.5 [d]		24	transient abs.	Konold 2019 [22]

[a] The two different lifetimes of S_1 (∼3 ps and ∼6-7 ps) were ascribed to structural heterogeneity of the carotenoid in OCP^O (at least two different carotenoid configurations are present) [16].
[b] Two different excitation wavelengths were used, namely 540 nm and 470 nm, giving rise to slightly different decay times (shorter for the 540-nm excitation) [16].
[c] As in Ref. [22], the two extracted times were ascribed to the S_1/ICT lifetimes of two different hECN populations in OCP, differing in hydrogen bonding via the carbonyl group [19].
[d] These two different decay times were both attributed to a state in which S_1 is coupled with an intramolecular charge-transfer (ICT) state, indicated as S_1/ICT [22].
[e] The ICT and S_1 names were assigned on the basis of the dominant contribution to the spectrum, although the two spectroscopic signatures contain both ICT and S_1 character [17, 21].

References

[1] Bondanza, M., Jacquemin, D. & Mennucci, B. Excited states of xanthophylls revisited: toward the simulation of biologically relevant systems. *J. Phys. Chem. Lett.* **12**, 6604–6612 (2021).

[2] Hui, B. *et al.* Detection of carotenoids on supercritical fluid chromatography (sfc). a preliminary investigation on the spectral shifts of carotenoids in supercritical carbon dioxide. *Chromatographia* **39**, 549–556 (1994).

[3] Chynwat, V. & Frank, H. A. Chemical physics elsevier the application of the energy gap law to the s 1 energies and dynamics of carotenoids. *Chemical Physics* **194**, 237–244 (1995).

[4] Bussi, G., Donadio, D. & Parrinello, M. Canonical sampling through velocity rescaling. *J. Chem. Phys.* **126**, 014101 (2007).

[5] Bondanza, M., Cupellini, L., Lipparini, F. & Mennucci, B. The multiple roles of the protein in the photoactivation of orange carotenoid protein. *Chem* **6**, 187–203 (2020).

[6] van Gunsteren, W. F. & Berendsen, H. J. C. Algorithms for brownian dynamics. *Mol. Phys.* **45**, 637–647 (1982).

[7] Cimiraglia, R., Malrieu, J.-P., Persico, M. & Spiegelmann, F. Quasi-diabatic states and dynamical couplings from ab initio ci calculations: a new proposal. *Journal of Physics B: Atomic and Molecular Physics* **18**, 3073 (1985).

[8] Persico, M. & Granucci, G. An overview of nonadiabatic dynamics simulations methods, with focus on the direct approach versus the fitting of potential energy surfaces. *Theoretical Chemistry Accounts* **133**, 1526 (2014).

[9] Persico, M. & Granucci, G. *Photochemistry: A modern theoretical perspective* (Springer, 2018).

[10] Accomasso, D., Persico, M. & Granucci, G. Diabatization by localization in the framework of configuration interaction based on floating occupation molecular orbitals (fomo-ci). *ChemPhotoChem* **3**, 933–944 (2019).

[11] Löwdin, P.-O. On the nonorthogonality problem. *Adv. Quantum Chem.* **5**, 185–199 (1970).

[12] Accomasso, D., Arslancan, S., Cupellini, L., Granucci, G. & Mennucci, B. Ultrafast excited-state dynamics of carotenoids and the role of the sx state. *J. Phys. Chem. Lett.* **13**, 6762–6769 (2022).

[13] D., C. & A., P. J. General definition of ring puckering coordinates. *J. Am. Chem. Soc.* **97**, 1354–1358 (1975).

[14] Chábera, P., Fuciman, M., Hříbek, P. & Polívka, T. Effect of carotenoid structure on excited-state dynamics of carbonyl carotenoids. *Phys. Chem. Chem. Phys.* **11**, 8795–8803 (2009).

[15] Khan, T. *et al.* Excited-state properties of canthaxanthin in cyanobacterial carotenoid-binding proteins HCP2 and HCP3. *J. Phys. Chem. B* **124**, 4896–4905 (2020).

[16] Šlouf, V. *et al.* Ultrafast spectroscopy tracks carotenoid configurations in the orange and red carotenoid proteins from cyanobacteria. *Photosynth. Res.* **131**, 105–117 (2017).

[17] Berera, R. *et al.* The photophysics of the orange carotenoid protein, a light-powered molecular switch. *J. Phys. Chem. B* **116**, 2568–2574 (2012).

[18] Niziński, S. *et al.* Unifying perspective of the ultrafast photodynamics of orange carotenoid proteins from synechocystis: Peril of high-power excitation, existence of different S^* states, and influence of tagging. *JACS Au* **2**, 1084–1095 (2022).

[19] Polívka, T., Kerfeld, C. A., Pascher, T. & Sundström, V. Spectroscopic properties of the carotenoid 3'-hydroxyechinenone in the orange carotenoid protein from the cyanobacterium arthrospira maxima. *Biochemistry* **44**, 3994–4003 (2005).

[20] Wilson, A. *et al.* A photoactive carotenoid protein acting as light intensity sensor. *Proc. Natl. Acad. Sci. U. S. A.* **105**, 12075–12080 (2008).

[21] Berera, R., Gwizdala, M., van Stokkum, I. H. M., Kirilovsky, D. & van Grondelle, R. Excited states of the inactive and active forms of the orange carotenoid protein. *J. Phys. Chem. B* **117**, 9121–9128 (2013).

[22] Konold, P. E. *et al.* Photoactivation mechanism, timing of protein secondary structure dynamics and carotenoid translocation in the orange carotenoid protein. *J. Am. Chem. Soc.* **141**, 520–530 (2019).
